# Photooxidation of 18‐Carbon Polyunsaturated Fatty Acids to Prepare Mono‐Hydroxylated Octadecanoids

**DOI:** 10.1002/lipd.70040

**Published:** 2026-02-17

**Authors:** Johanna Revol‐Cavalier, Mats Hamberg, Craig E. Wheelock

**Affiliations:** ^1^ Unit of Integrative Metabolomics Institute of Environmental Medicine, Karolinska Institutet Stockholm Sweden; ^2^ Larodan Research Laboratory Karolinska Institutet Stockholm Sweden; ^3^ Department of Respiratory Medicine and Allergy Karolinska University Hospital Stockholm Sweden

**Keywords:** octadecanoids, octadecapentaenoic acid, photosensitized oxidation, polyunsaturated fatty acids, singlet oxygen

## Abstract

Oxylipins are oxygenated products of fatty acids that are being increasingly studied due to their role in multiple physiological processes. Investigations to date have focused on the 20‐carbon eicosanoids and 22‐carbon docosanoids. However, more recently, interest has grown into the 18‐carbon octadecanoids. A significant obstacle in the study of these compounds is a lack of authentic standards for functional studies as well as for development of methods for their quantification. We developed a fast and simple one‐step synthetic strategy to produce mono‐hydroxylated metabolites based on the photosensitized oxidation of 18‐carbon polyunsaturated fatty acids (PUFAs). Four different PUFAs including α‐linolenic acid (ALA, ω3), γ‐linolenic acid (GLA, ω6), stearidonic acid (SDA, ω3), and octadecapentaenoic acid (ODPA, ω3) were photooxidized in the presence of methylene blue and oxygen under exposure to light from a halogen lamp. The uncommon PUFA ODPA was prepared from docosahexaenoic acid (DHA, ω3) in a 6‐step synthesis with 30% overall yield. The hydroperoxide products were reduced with sodium borohydride and the mono‐hydroxylated octadecanoids were separated by HPLC. Product identification was performed by GC–MS. The final purities of isolated products ranged from 80% to 98%, with oxidation of non‐terminal double bonds being preferred. It is likely that this approach could be extended to PUFAs of varying chain length, suggesting that photosensitized oxidation could be employed to rapidly prepare hydroperoxides from multiple unsaturated fatty acids. As interest in oxylipins continues to increase, this approach will be useful for large‐scale preparation of multiple standards for the study of these new compounds.

## Graphical abstract

We developed a fast and simple one‐step synthetic strategy to produce mono‐hydroxylated metabolites based on the photosensitized oxidation of 18‐carbon polyunsaturated fatty acids (PUFAs). This approach could likely be extended to other PUFAs, suggesting that photosensitized oxidation could be employed to rapidly prepare hydroperoxides from multiple unsaturated fatty acids.

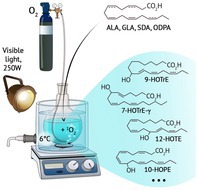

## 1 Introduction

Oxylipins are oxygenated products of mono‐ and polyunsaturated fatty acids (PUFA) (Gerwick et al. 1991). Multiple molecules from this class have been shown to exert functionality in a range of biological processes (Parchem et al. 2024). A more recently defined sub‐group includes the oxylipins derived from 18‐carbon fatty acids, which have been termed octadecanoids (Revol‐Cavalier et al. 2025). Less is known about octadecanoids relative to their 20‐carbon (eicosanoids) or 22‐carbon (docosanoids) analogs. One of the reasons for this paucity of information is a lack of chemical standards for functional assays or development of analytical methods. Accordingly, there is a need for methods to synthesize multiple PUFA‐derived octadecanoids that have not been previously studied. While protocols for the production of enzymatically‐derived octadecanoid hydroperoxides in the 9‐carbon (Gardner and Grove 2001) and 13‐carbon positions (Iacazio et al. 1990) have been published, there is a lack of concerted methods for broad‐scale production of multiple mono‐hydroxy compounds.

**SCHEME 1:**
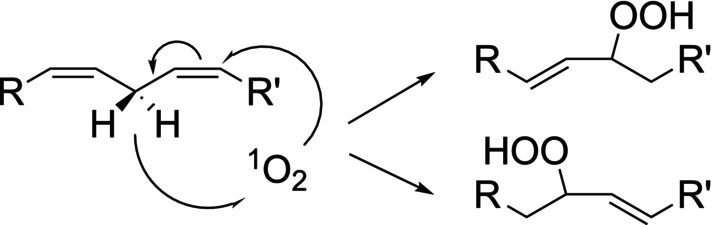
Formation of hydroperoxides by singlet oxygen.

Triplet (ground state) dioxygen participates in radical reactions because of its two unpaired electrons; however, oxygen in the singlet state (^1^O_2_) has these electrons paired in a single orbital and therefore enters non‐radical reactions. ^1^O_2_ is strongly electrophilic and typically reacts with carbon–carbon double bonds to produce endoperoxides or hydroperoxides. Hydroperoxides are formed by an ene‐reaction in which the singlet oxygen molecule attacks one of the alkene carbons and one of the hydrogens on the allylic position (Frimer 1979) (Scheme 1). This process has been demonstrated to have suprafacial sterochemistry (Alberti et al. 2008), and so does the reaction of singlet oxygen with PUFAs (Hamberg 2011).

Singlet oxygen can be generated by performing oxygen‐releasing reactions in which the O_2_ molecule emerges in the singlet state. Such reactions include oxidation of hydrogen peroxide with hypochlorite (Held et al. 1978), thermal decomposition of endoperoxides (Wasserman et al. 1972) and ozonides (Murray and Kaplan 1969), and decomposition of highly oxidized metal salts such as potassium perchromate (Peters et al. 1975). Alternatively, ^1^O_2_ can be produced by bubbling ground‐state oxygen into solutions containing light‐excited photosensitizers such as methylene blue and rose Bengal (Foote 1968).

Singlet oxygen is formed in living tissues where it contributes to fatty acid hydroperoxide formation along with lipoxygenases and autoxidation. In plants, ^1^O_2_ is formed from ground‐state oxygen when interacting with light‐generated triplet‐state chlorophyll (Triantaphylidès and Havaux 2009). Because of its extremely short half‐life (200 ns; Gorman and Rodgers 1992), ^1^O_2_ will react only with PUFAs that are located in close proximity to its site of formation. Tissue damage can result from the hydroperoxides generated, either by direct oxidation or by way of reactive oxygen species formed by metal‐induced decomposition of the hydroperoxides. Singlet oxygen has been proposed to have a signaling role in plants (Przybyla et al. 2008). In animals, singlet oxygen is formed in reactions catalyzed by NADPH oxidase and myeloperoxidase. This process can result in tissue damage; however, generation of singlet oxygen in polymorphonuclear leukocytes serves a protective role by killing phagocytized bacteria (Steinbeck et al. 1992).

There have been several studies performed on photosensitized oxygenations of PUFAs of varying alkyl chain length. Thomas and Pryor photooxidized methyl linoleate using methylene blue as a sensitizer followed by NaBH_4_ reduction of the hydroperoxides (Thomas and Pryor 1980). Roughly equal amounts of the 9‐, 10‐, 12‐, and 13‐hydroxy‐octadecadienoates were formed. In 2006, Zhang et al. (2006) further studied the photosensitized oxidation of methyl linoleate and showed that irradiation with a tungsten lamp in the presence of tetraphenylporphine resulted in the primary formation of monohydroperoxides (70% yield) in a mixture with 3 dihydroperoxides (9,12; 10,12; and 10,13, collectively 12% yield). Arachidonic acid was photooxidized by methylene blue to obtain the 8 possible regioisomeric hydroperoxides as well as further oxygenated peroxides (Terao and Matsushita 1981). The longer chain docosahexaenoic acid was photooxidized to produce the 12 possible regioisomeric oxygenated products, both as hydroperoxides and as hydroxides in a NaBH_4_‐reduced sample (Derogis et al. 2013).

In the current study, we performed a systematic investigation of the photooxidation of four 18‐carbon PUFAs: α‐linolenic acid (18:3ω3, ALA), γ‐linolenic acid (18:3ω6, GLA), and stearidonic acid (18:4ω3, SDA) as well as the uncommon octadecapentaenoic acid (18:5ω3, ODPA). The products were treated with NaBH_4_, and the resulting hydroxides were separated by normal‐phase HPLC and identified by GC–MS. These efforts successfully produced several of the mono‐hydroxy products of these C18 PUFAs, providing an effective way to produce these oxylipins for future investigations.

## 2 Materials and Methods

### 2.1 Photosensitized Oxidation of PUFAs

**SCHEME 2:**
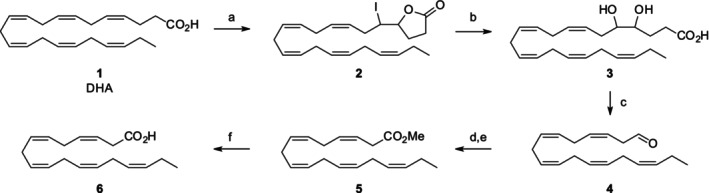
Synthesis of ODPA **6** from DHA **1**. (a) 2,4,6‐trimethylpyridine, Iodine, CH_3_CN. (b) LiOH, THF/H_2_O. (c) AcOH, NaIO_4_, Acetone/H_2_O. (d) t‐BuOH, 2‐methyl‐2‐butene, NaH_2_PO_4_, NaClO_2_. (e) CH_2_N_2_, MeOH. (f) LiOH, THF/H_2_O.

All reagents and solvents were obtained from Merck (Missouri, US) and TCI (Tokyo, Japan) and used without further purification. The PUFAs α‐linolenic acid (ALA, 18:3), γ‐linolenic acid (GLA, 18:3), and stearidonic acid (SDA, 18:4) were obtained from Larodan (Stockholm, Sweden). Octadecapentaenoic acid (ODPA, 18:5) was synthesized in house from DHA (Scheme 2). All PUFAs were checked for purity by GC–MS before use.

#### 2.1.1 Synthesis of ODPA

The OPDA has been obtained in 6 steps of synthesis following Scheme 2:

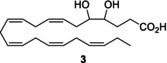

##### 2.1.1.1 4,5‐Dihydroxy‐7(*Z*),10(*Z*),13(*Z*),16(*Z*),19(*Z*)‐Docosapentaenoic Acid (**3**)

To a stirred mixture of DHA **1** (1 g, 3.05 mmol, 1 equiv.) in 104 mL of acetonitrile containing 2,4,6‐trimethylpyridine (1.7 mL, 12.9 mmol, 4.2 equiv.) at 0°C was added iodine (1.6 g, 6.3 mmol, 2.06 equiv.). After stirring under nitrogen at 0°C for 15 min, the mixture was warmed to room temperature and stirred for 2.5 h. Water was added and the aqueous layer was extracted with ethyl acetate. The organic layer was washed successively with NaHSO_3_ solution, 0.5 M HCl and water and dried over MgSO_4_ affording the iodolactone **2**. MS (EI+, 70 eV): *m*/*z* 454 [M]^+^, 327 [M‐I]^+^, 175 and 105.

The crude iodolactone **2** was dissolved in 100 mL of tetrahydrofuran, cooled to 0°C and treated with a solution of LiOH (800 mg, 33.3 mmol, 11 equiv.) in 67 mL of water. The mixture was stirred at room temperature for 3 h and subsequently carefully acidified to pH 3 with HCl 1 M and extracted with ethyl acetate to afford the diol **3** (977 mg, 2.7 mmol, 89% yield, 2 steps). MS (EI+, 70 eV) of the trimethylsilyl (Me_3_Si) ester/methyl ether derivative: *m*/*z* 578 [M]^+^, 473 [M‐(CH_3_ + Me_3_SiOH)]^+^, 349 [Me_3_SiO^+^ = CH‐CH(OSiMe_3_)‐CH_2_‐CH_2_‐COOSiMe_3_], and 247 [Me_3_SiO^+^ = CH‐CH_2_‐CH_2_‐COOSiMe_3_].

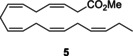

##### 2.1.1.2 Methyl 3(*Z*),6(*Z*),9(*Z*),12(*Z*),15(*Z*)‐Octadecapentaenoate (**5**)

Diol **3** (977 mg, 2.7 mmol, 1 equiv.) was dissolved in 128 mL of acetone and 32 mL of water was added. The solution was cooled to 0°C and glacial acetic acid (9.7 mL) followed by NaIO_4_ (3.8 g, 17.8 mmol, 6.6 equiv.) were added under stirring. The mixture was warmed to room temperature and stirred for 1 h. Water was added, and the aqueous layer was extracted with hexanes. The organic layer was dried over MgSO_4_ and concentrated under vacuum to afford crude aldehyde **4** as a light‐brown oil. MS (EI+, 70 eV): *m*/*z* 257 [M‐H]^+^, 243 [M‐CH_3_]^+^, 229 [M‐CH_2_CH_3_]^+^, 105, 91 and 78.

Part of this material (300 mg, 1.16 mmol, 1 equiv.) was directly subjected to sodium chlorite oxidation as described by Lindgren and Nilsson (Fiasella et al. 2014; Lindgren et al. 1973). Thus, the material was dissolved in 44 mL of 79% aqueous *tert*‐BuOH containing 2‐methyl‐2‐butene (9 mL) and NaH_2_PO_4_ (489 mg, 4 mmol, 3.4 equiv.) and the mixture was stirred at room temperature for 15 min. Subsequently, NaClO_2_ (968 mg, 10.7 mmol, 9.2 equiv.) was added and after further stirring for 15 min, water was added, and the aqueous layer was extracted with diethyl ether. The organic layer was dried over MgSO_4_, concentrated under vacuum and dissolved in methanol. An ethereal solution of diazomethane (Ngan and Toofan 1991) was added to afford the crude title compound (301 mg, purity > 90%). GC–MS analysis revealed a major peak of **5** and showed no trace of **4** remaining unoxidized. However, several later eluting peaks due to chloro‐hydrin adducts of **5** were also present. These adducts were removed by purification by reversed‐phase HPLC (CH_3_CN‐H_2_O [75:25, v/v]) followed by silica gel chromatography (isopropanol/hexane [2:98, v/v]). The title compound was obtained as a colorless oil (111 mg, 0.39 mmol, purity > 98%, yield 34%).

MS (EI+, 70 eV): *m*/*z* 288 [M]^+^, 259 [M‐CH_2_CH_3_]^+^, 201 [M‐CH_2_COOCH_3_]^+^, 175, 145 and 105.

^1^H NMR (400 MHz, Chloroform‐*d*) δ 5.62–5.50 (m, 2H, MeO_2_CCH_2_C*H*=C*H*), 5.43–2.25 (m, 8H, C*H*=C*H*), 3.67 (s, 3H, C*H*_3_O), 3.15–3.06 (m, 2H, MeO_2_CC*H*_2_), 2.88–2.76 (m, 8H, C*H*_2_), 2.07 (q, *J* = 7.3 Hz, 2H, C*H*_2_CH_3_), 0.95 (t, *J* = 7.5 Hz, 3H, CH_2_C*H*_3_) (Scheme S6).

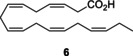

##### 2.1.1.3 3(*Z*),6(*Z*),9(*Z*),12(*Z*),15(*Z*)‐Octadecapentaenoic Acid (**6**)

Because of the homoallylic double bond in **5**, its hydrolysis into **6** required very mild conditions. Thus, **5** (106 mg, 0.35 mmol, 1 equiv.) was dissolved in 19 mL of tetrahydrofuran and cooled to 0°C. A precooled solution of LiOH (46 mg, 1.9 mmol, 5.4 equiv.) in 19 mL of water was added under stirring and stirring continued for 5 min. Subsequently, the solution was warmed to room temperature, and the mixture was stirred for further 45 min. Careful acidification and extraction with ethyl acetate afforded the title compound as a colorless oil in essentially quantitative yield. It was established by GC–MS that no *trans* or positional double bond isomers were obtained.

#### 2.1.2 General Procedure for the Photosensitized Oxidation of PUFAs

In a 250‐mL flask, the fatty acid (100 mg) was dissolved in methanol (50 mL). Methylene blue (25 mg) was added, and oxygen was bubbled into the reaction. The reaction was stirred for 7 h at 6°C under exposure of a 250‐W lamp diffusing visible light (Philips projection lamp type 1316). After a full conversion was observed by GC–MS, the reaction was cooled at 0°C, and NaBH_4_ was added until the disappearance of the blue color. The reaction was stirred for 30 min, quenched with H_2_O and acidified to pH 2 with 1 M HCl. The layers were separated, and the aqueous layer was extracted 3× with diethyl ether. The organic layer was washed with brine, dried over MgSO_4_, filtered, and concentrated under vacuum. The crude oil was purified on a Buchi flash chromatography system (silica column: 20 g, gradient: 98/2 to 88/12 Hexanes/(iPrOH + CH_3_CO_2_H, 0.1%), 20 column volumes) to afford the mixture of mono‐hydroxylated metabolites.

#### 2.1.3 Separation of the Isomers by HPLC

The different regioisomers were separated on normal phase HPLC (possessing a Spectra series P100 isocratic pump and a Spectra UV 100 detector from Thermo Separation Products, Fremont, CA, and a refractive index model 8120P detector from Bischoff Chromatography, Leonberg, Germany and operated with the McDAD 100 chromatographic software purchased from Bischoff Chromatography, Leonberg, Germany). A 250 × 10 mm column of SP‐250/10 Nucleosil 50–7 purchased from Marcherey‐Nagel (Düren, Germany) was used at a flow rate of 4 mL/min; mobile phases, iPrOH/hexane/CH_3_CO_2_H 1.5/98.5/0.01 (v/v/v) for ALA, GLA, and SDA metabolites and iPrOH/hexane/CH_3_CO_2_H 1.2/98.8/0.01 (v/v/v) for ODPA metabolites. Detection was performed using either UV (210 nm) or refractive index (RI). The mixtures of 15‐hydroxy‐octadecatrienoic acid (15‐HOTrE) and 16‐HOTrE were purified on reversed‐phase HPLC (possessing a HPLC Compact pump 2250 and a refractive index model 8120P detector from Bischoff Chromatography, Leonberg, Germany, and a Spectromonitor III UV detector from Laboratory Data Control, operated with the software scintflow B2 V.3.03) using a 250 × 4.6 mm column of EC 250/4 Nucleosil 100–5 C18 at a flow rate of 2 mL/min; mobile phase, CH_3_CN/H_2_O/CH_3_COOH 40/60/0.01 v/v/v and monitored using either UV (210 nm) or RI.

#### 2.1.4 Analysis of the Mono‐Hydroxylated Products

The free acids (1 mg) were solubilized in methanol (100 μL) and derivatized into the methyl ester with the dropwise addition of a freshly prepared solution of diazomethane in diethyl ether (prepared from Diazald according to the manufacturer's instructions; Ngan and Toofan 1991). As soon as a persistent yellow color was observed, the solvents were removed under a stream of nitrogen and the free alcohols were derivatized into TMS ether with a mixture of TMSCl/pyridine/hexamethyldisilazane (v/v/v 2/2/1) for 15 min, dried under vacuum, diluted in hexanes, centrifuged, and injected. The obtained products were analyzed by an Agilent 5977 GC/MSD (Santa Clara, CA, USA) using a 12 m HP‐ultra‐1 column (Agilent J&W GC columns) with 0.33‐μm film of 5% phenylmethylsiloxane, using helium as carrier gas. Injector temperature 200°C. Injection: 0.5 μL. Temperature program: 120°C–320°C, 10°C/min.

## 3 Results and Discussion

**SCHEME 3:**
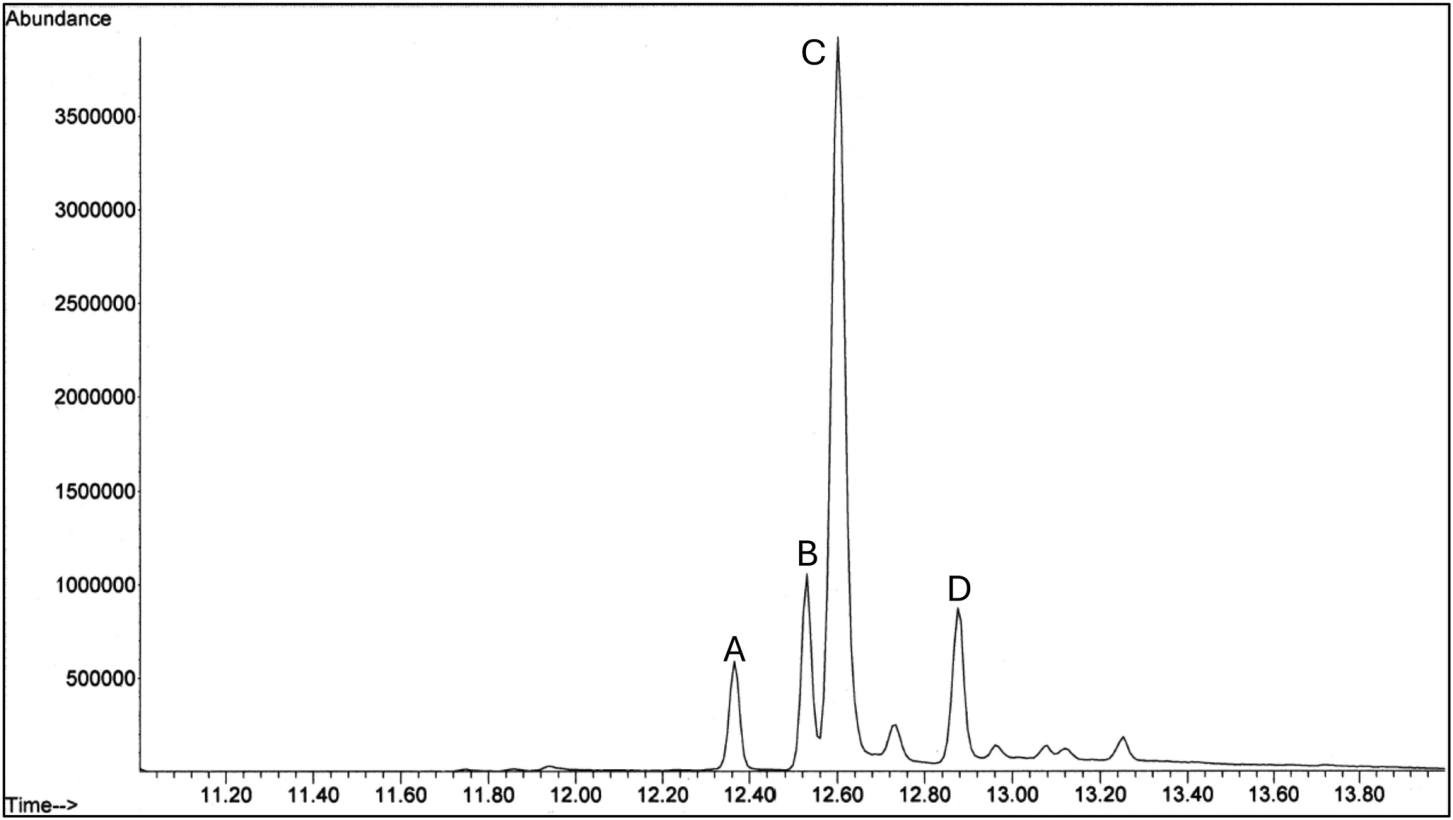
GC–MS chromatogram of the photosensitized oxidation of α‐linolenic acid (ALA). Peak A = 10‐HOTrE, Peak B = 15‐HOTrE, Peak C = 12‐HOTrE, 13‐HOTrE, and 9‐HOTrE, Peak D = 16‐HOTrE. HOTrE = hydroxy‐octadecatrienoic acid.

The result of the photosensitized oxidation of ALA by GC–MS is depicted in Scheme 3. The TMS derivatives of the methyl esters of Hydroxy‐OctadecaTriEnoic acids (HOTrEs) eluted under four peaks (A, B, C, D) with retention times between 12.20 and 13.00 min.

**SCHEME 4:**
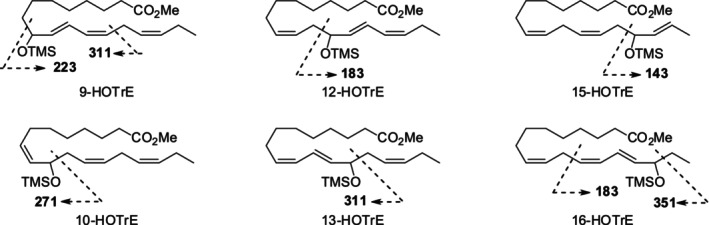
Fragmentation of hydroxy‐octadecatrienoic acids (HOTrEs) from α‐linolenic acid (ALA).

According to the ene‐reaction mechanism, the photosensitized oxidation of ALA gave 6 mono‐hydroxylated products (9‐, 10‐, 12‐, 13‐, 15‐, and 16‐HOTrEs). The peak assignment was made based on MS fragments. The fragment ion resulting in cleavage of the α‐position of the trimethylsilyloxyl (OTMS) was formed at *m*/*z* 223 for methyl 9‐hydroxy‐octadecatrienoate, *m*/*z* 271 for the 10‐isomer, *m*/*z* 183 for the 12‐isomer, *m*/*z* 311 for the 13‐isomer, *m*/*z* 143 for the 15‐isomer, and *m*/*z* 351 for the 16‐isomer. Additionally, the fragments resulting from α‐cleavage of the conjugated *cis*‐double bond were observed for the two outer isomers and gave rise to *m*/*z* 311 for the TMS‐derivative of methyl 9‐hydroxy‐octadecatrienoate and *m*/*z* 183 for the TMS‐derivative of methyl 16‐hydroxy‐octadecatrienoate (Scheme 4).

Based on the *m*/*z* ratios, it was determined that peak A contained the methyl ester of 10‐HOTrE (8%), and peak B contained the methyl ester of 15‐HOTrE (13.7%), while peak C constituted the methyl esters of 12‐HOTrE, 13‐HOTrE, and 9‐HOTrE (73%), with 9‐HOTrE eluting exclusively at the end of the peak. Finally, peak D was determined to be the methyl ester of 16‐HOTrE (5.3%) (Scheme 3). Under these conditions, it appeared that the oxidation of the inner double bond was preferred. This observation is in opposition to a previous study where the authors oxidized esterified ALA at 0°C in methanol (10 mL/2 g ester) in the presence of methylene blue (9 mg/2 g ester), under exposure to a 1000 W tungsten light source for 2 h. They reported a ratio favoring the outer hydroperoxides, with about 23%–25% of 9‐ and 16‐hydroperoxides formed against 12%–14% of the inner isomers: 10‐, 12‐, 13‐, and 15‐hydroperoxides (Frankel et al. 1979). Possibly, the light intensity and the reaction time influenced the ratio of metabolites obtained, with a higher light intensity (1000 vs. 250 W) coupled with a shorter reaction time (2 vs. 7 h) favoring formation of the outer hydroperoxides.

**SCHEME 5:**
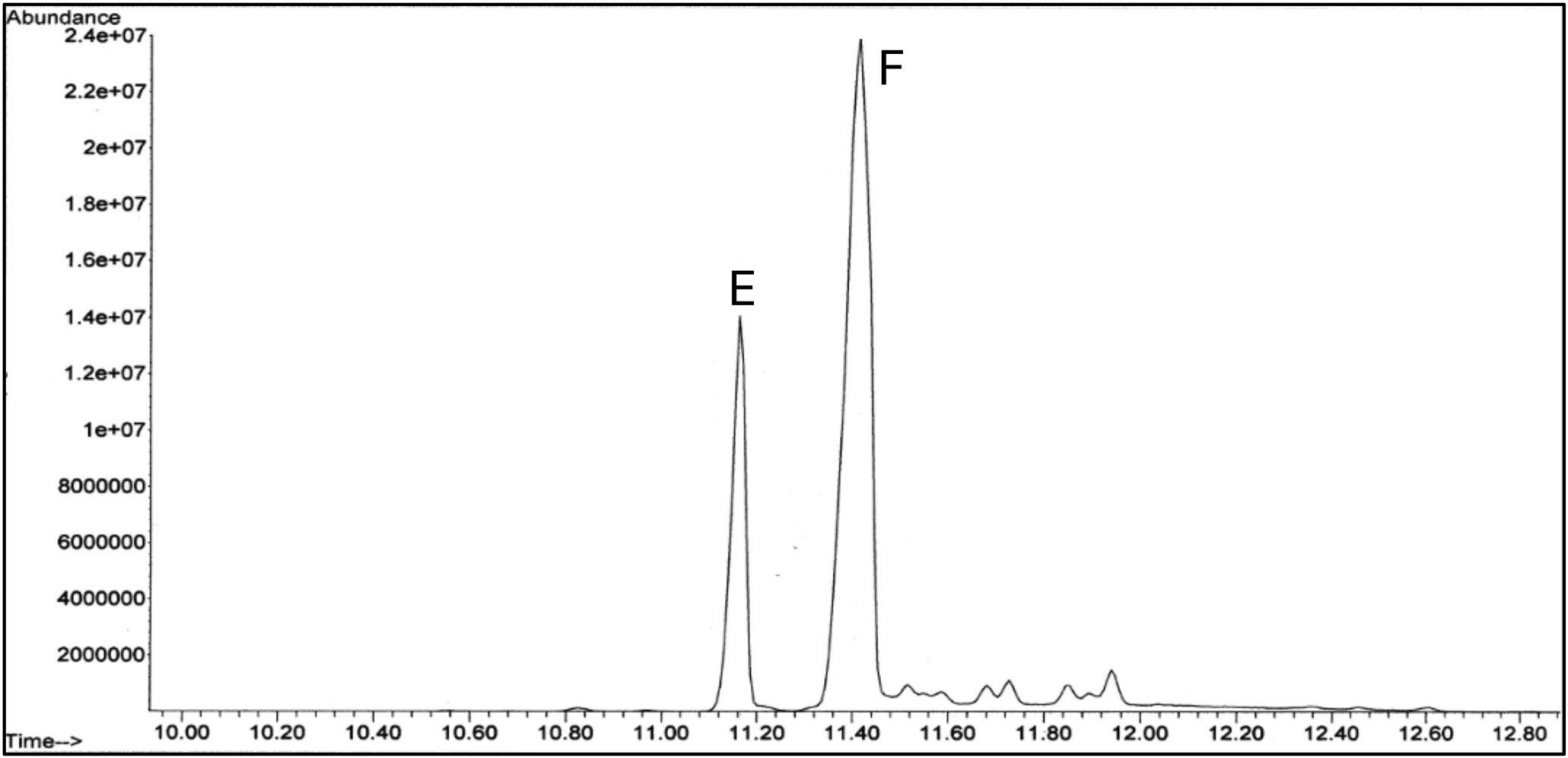
GC–MS chromatogram of the photosensitized oxidation of γ‐linolenic acid (GLA). Peak E = 7‐HOTrE‐γ and 12‐HOTrE‐γ, Peak F = 9‐HOTrE‐γ, 10‐HOTrE‐γ, and 13‐HOTrE‐γ. HOTrE = hydroxy‐octadecatrienoic acid.

After derivatization, the photosensitized oxidation of GLA gave two peaks (E and F), with retention times between 11.00 and 11.50 min (Scheme 5).

**SCHEME 6:**
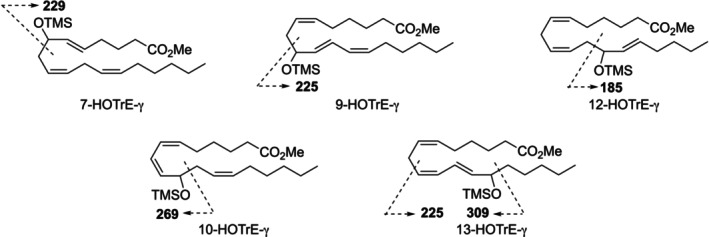
Fragmentation of hydroxy‐octadecatrienoic acids (HOTrEs‐γ) from γ‐linolenic acid (GLA).

The fragment ions resulting from cleavage of the α‐position of the trimethylsilyloxyl group were formed at *m*/*z* 229 for methyl 7‐hydroxy‐octadecatrienoate, *m*/*z* 225 for the 9‐isomer, *m*/*z* 185 for the 12‐isomer, and *m*/*z* 309 for the 13‐isomer. Additionally, the fragment resulting from α‐cleavage of the conjugated *cis*‐double bond was observed for the outer isomer 6(*Z*),9(*Z*),11(*E*)‐13‐HOTrE (or 13‐HOTrE‐γ) methyl ester and gave rise to *m*/*z* 225 (Scheme 6).

Analysis of the mass spectra indicated that only 5 products were formed during the reaction, because the product of the ene‐reaction on position 6, namely 6‐HOTrE‐γ, was not detected. The absence of this product could be explained by a rapid further oxidation leading to a diol formation, or a very low abundance. Peak E contained the methyl esters of 7‐HOTrE‐γ and 12‐HOTrE‐γ (25.5%), and peak F contained the methyl esters of 9‐HOTrE‐γ, 10‐HOTrE‐γ, and 13‐HOTrE‐γ (74.5%) (Scheme 5).

**SCHEME 7:**
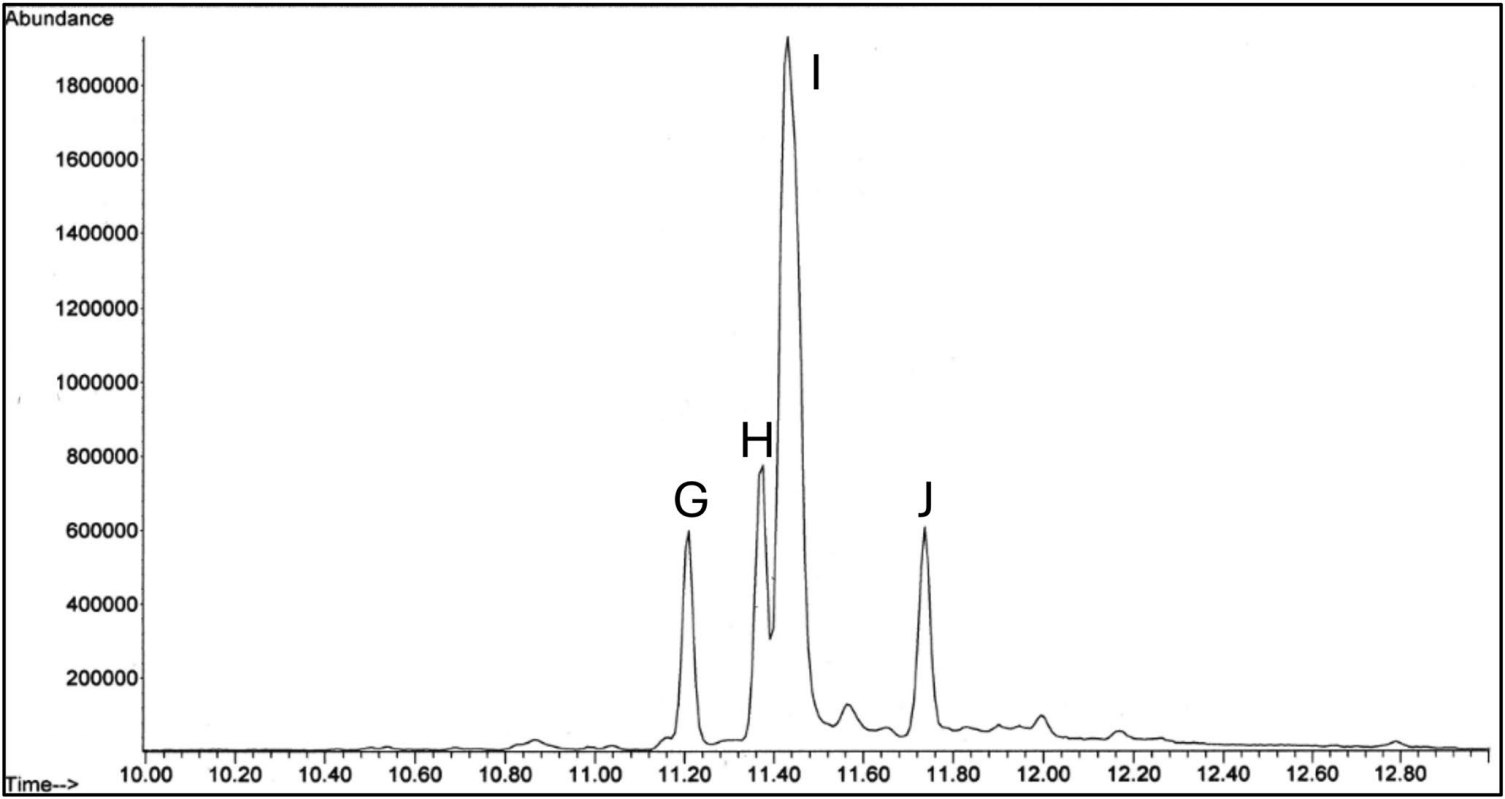
GC–MS chromatogram of the photosensitized oxidation of stearidonic acid (SDA). Peak G = 7‐HOTE, Peak H = 15‐HOTE, Peak I = 9‐HOTE, 10‐HOTE, 12‐HOTE and 13‐HOTE, Peak J = 16‐HOTE. HOTE = hydroxy‐octadecatetraenoic acid.

Scheme 7 shows the GC–MS result of the photosensitized oxidation of SDA, which gave 4 peaks (G, H, I, J) by GC–MS with retention times between 11.00 and 12.00 min.

**SCHEME 8:**
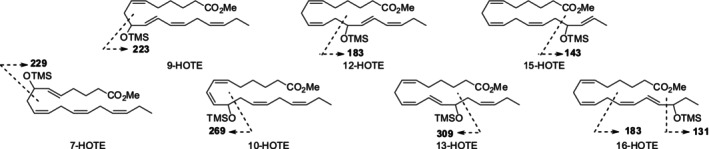
Fragmentation of hydroxy‐octadecatranoic acids (HOTEs) from stearidonic acid (SDA)

The fragment ions resulting from cleavage at the α‐position of the trimethylsilyloxyl group were formed at *m*/*z* 229 for methyl 7‐hydroxy‐octadecatetraenoate, *m*/*z* 223 for the 9‐isomer, *m*/*z* 269 for the 10‐isomer, *m*/*z* 183 for the 12‐isomer, *m*/*z* 309 for the 13‐isomer, *m*/*z* 143 for the 15‐isomer, and *m*/*z* 183 for the 16‐isomer. Additionally, the fragment resulting from cleavage at the α‐position of the conjugated *cis*‐double bond was observed for the outer isomer 16‐Hydroxy‐OctadecaTetraEnoic acid (16‐HOTE) methyl ester and was formed at *m*/*z* 131 (Scheme 8).

Similarly to GLA oxidation, it was observed that only 7 products were formed in the reaction, with 6‐HOTE not abundant enough to be detected. It is unclear why oxidation at the 6‐position was consistently not observed for GLA and SDA. One possible explanation is that the rate of further dioxygenation of the 6‐hydroperoxides was higher than those for the other hydroperoxide isomers. Peak G was determined to be the methyl ester of 7‐HOTE (11.5%), peak H contained the methyl ester of 15‐HOTE (15.2%), and peak I consisted of the methyl esters of 9‐HOTE, 10‐HOTE, 12‐HOTE, and 13‐HOTE (62.7%). A small separation of the two 10‐/12‐hydroxy and the two 9‐/13‐hydroxy isomers was noted, with the two former isomers being enriched in the first half of peak I and the latter ones in the second half of the peak. Finally, compound J was the methyl ester of 16‐HOTE (10.6%) (Scheme 7).

**SCHEME 9:**
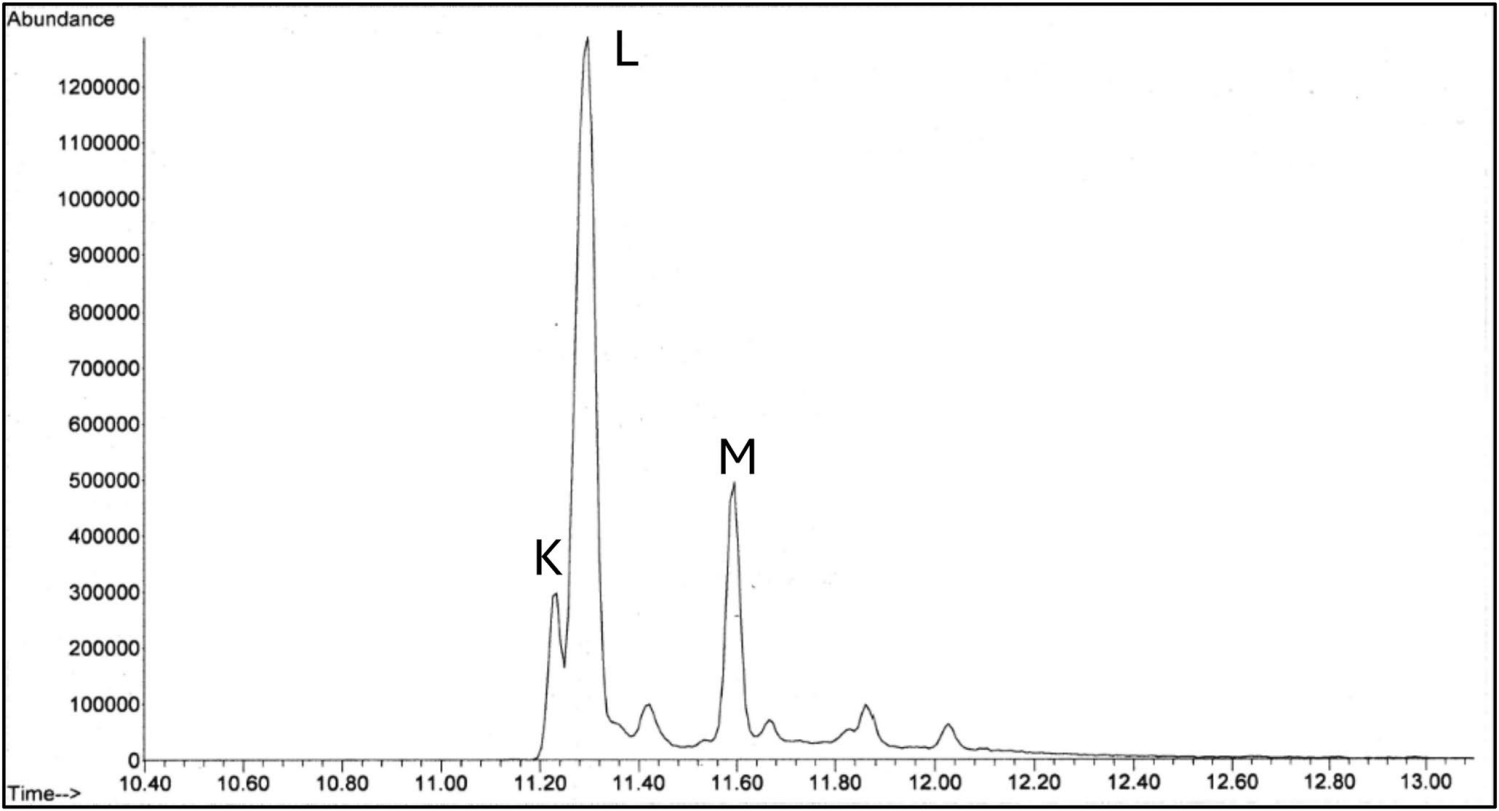
GC–MS chromatogram of the photosensitized oxidation of octadecapentaenoic acid (ODPA). Peak K = 15‐HOPE, Peak L = 9‐HOPE, 10‐HOPE, 12‐HOPE and 13‐HOPE, Peak M = 16‐HOPE. HOPE = hydroxy‐octadecapentaenoic acid.

ODPA oxidation occurred faster than the oxidation of the other PUFAs, with primarily diols obtained after 16 h of reaction. The reaction time was decreased to 7 h; however, a large proportion of diols (37%) was still obtained (the reaction was checked after 3 h and only 48% of conversion was observed). It is possible that the reaction time could be further optimized between 3 and 7 h to increase overall yields. The diols could be completely separated from the mono‐hydroxylated metabolites by flash chromatography (and were not further purified), and 16 mg of the mixture of mono‐hydroxylated metabolites was obtained. The GC–MS profile of the mono‐hydroxy products formed from ODPA is shown in Scheme 9. Three main peaks (K, L, M) containing the expected hydroxy‐octadecapentaenoic acids (HOPEs) were observed by GC–MS with retention times between 11 and 12 min.

**SCHEME 10:**
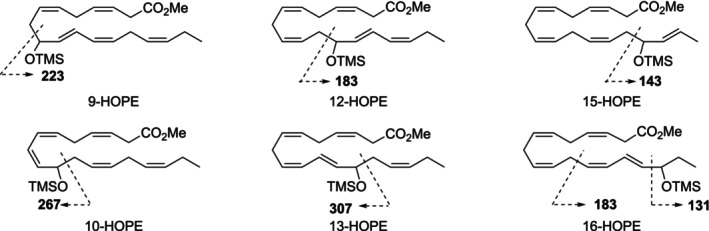
Fragmentation of hydroxy‐octadecapentaenoic acids (HOPEs) from octadecapentaenoic acid (ODPA).

The fragment ions resulting from cleavage at the α‐position of the trimethylsilyloxyl group were formed at *m*/*z* 223 for the 9‐isomer, *m*/*z* 267 for the 10‐isomer, *m*/*z* 183 for the 12‐isomer, *m*/*z* 307 for the 13‐isomer, *m*/*z* 143 for the 15‐isomer, and *m*/*z* 183 for the 16‐isomer. Additionally, the fragment resulting from cleavage at the α‐position of the conjugated *cis*‐double bond was observed for the outer isomer 16‐HOPE methyl ester and was formed at *m*/*z* 131 (Scheme 10).

Only 6 products were obtained, and the products of the ene‐reaction on position 3, 4, 6, and 7, namely 3‐HOPE, 4‐HOPE, 6‐HOPE, and 7‐HOPE, could not be detected. It is quite surprising that the 7‐position was not oxidized on ODPA, because attack at this position by singlet oxygen was observed for GLA and SDA, and the formation of the resulting 7‐HOTrE‐γ and 7‐HOTE was observed. Peak K contained the methyl ester of 15‐HOPE (10%), peak L contained the methyl esters of 9‐HOPE, 10‐HOPE, 12‐HOPE, and 13‐HOPE (70%), and peak M contained the methyl ester of 16‐HOPE (20%) (Scheme 9).

**SCHEME 11:**
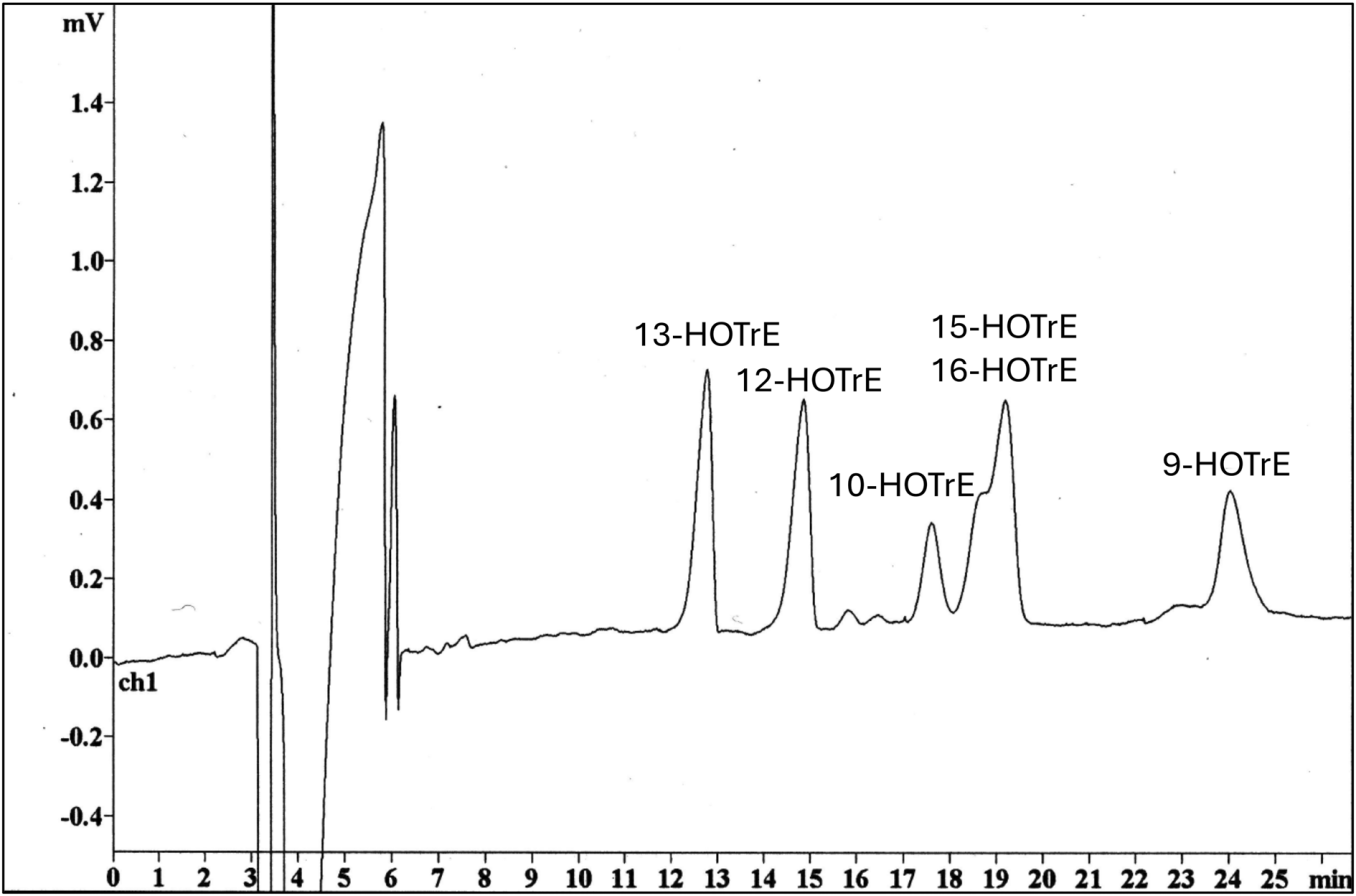
HPLC separation of the mixture of mono‐hydroxylated metabolites of ALA (RI detector).

The 4 mixtures of mono‐hydroxylated metabolites were purified on normal‐phase HPLC. The eluent used for the separation of the metabolites of ALA, GLA, and SDA was a mixture of iPrOH/hexanes 1.5/98.5 + CH_3_CO_2_H 0.01%. To separate the ODPA metabolites, the polarity had to be reduced, and a mixture of iPrOH/hexanes 1.2/98.8 + CH_3_CO_2_H 0.01% was used. All ALA metabolites had a retention time between 12.00 and 25.00 min (Scheme 11). Each of the collected peaks were analyzed by GC–MS to confirm identity and purity (Scheme S2).

**SCHEME 12:**
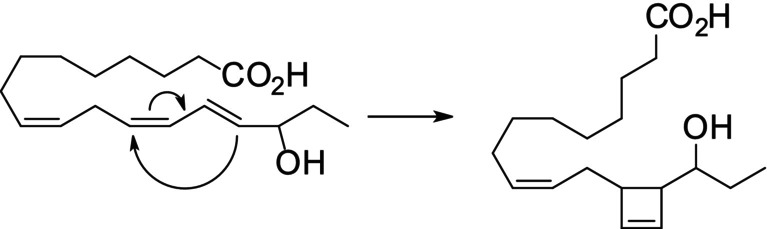
Proposed explanation for the formation of a tail by GC–MS for the two outer hydroxyls.

15‐HOTrE and 16‐HOTrE were further separated on RP‐HPLC, and a separation was observed by a refractive index detector (RT = 28.30 min for 15‐HOTrE, RT = 30.50 min for 16‐HOTrE) (Scheme S1). Except for 15‐HOTrE, which was obtained in mixture with 8% of 16‐HOTrE, ALA metabolites were obtained with good (> 94%) to very high (> 98%) purities (Table 1). The two outer hydroxyls, 9‐ and 16‐HOTrEs, had tailing peaks by GC–MS. We hypothesize that this was due to a rearrangement at high temperature to form a cyclobutene (Scheme 12).

**TABLE 1:** Purity and amount of the purified octadecanoids.

Parent PUFA	Octadecanoid	Amount	Purity (GC–MS)	Yield
ALA	9‐HOTrE	5.3 mg	95%	5.0%
	10‐HOTrE	4.4 mg	94%	4.2%
	12‐HOTrE	8.4 mg	> 98%	7.9%
	13‐HOTrE	7.4 mg	> 98%	7.0%
	15‐HOTrE	1.7 mg	91%	1.6%
	16‐HOTrE	2.9 mg	97%	2.7% Global yield: 28.4%
GLA	7‐HOTrE‐γ	0.7 mg	> 98%	0.7%
	9‐HOTrE‐γ	7.3 mg	96%	6.9%
	10‐HOTrE‐γ	8.1 mg	95%	7.6%
	12‐HOTrE‐γ	4.5 mg	> 98%	4.3%
	13‐HOTrE‐γ	7.5 mg	97%	7.1% Global yield: 26.6%
SDA	9‐HOTE	2.2 mg	80%	2.1%
	10‐HOTE	2.3 mg	> 98%	2.2%
	12‐HOTE	1.9 mg	98%	1.8%
	13‐HOTE	3.0 mg	98%	2.9%
	15‐HOTE	2.1 mg	98%	2.0%
	16‐HOTE	2.5 mg	95%	2.4% Global yield: 13.4%
OPDA	9‐HOPE	3.4 mg	80%	3.2%
	10‐HOPE	1.3 mg	> 98%	1.2%
	12‐HOPE/15‐HOPE	0.7 mg	60/40, 94%	0.7%
	13‐HOPE	1.0 mg	> 98%	0.9%
	16‐HOPE	Degraded	Degraded	NA Global yield: 6.0%

Abbreviation: NA = not applicable, the 16‐HOPE was not stable and therefore no yield is reported.

**SCHEME 13:**
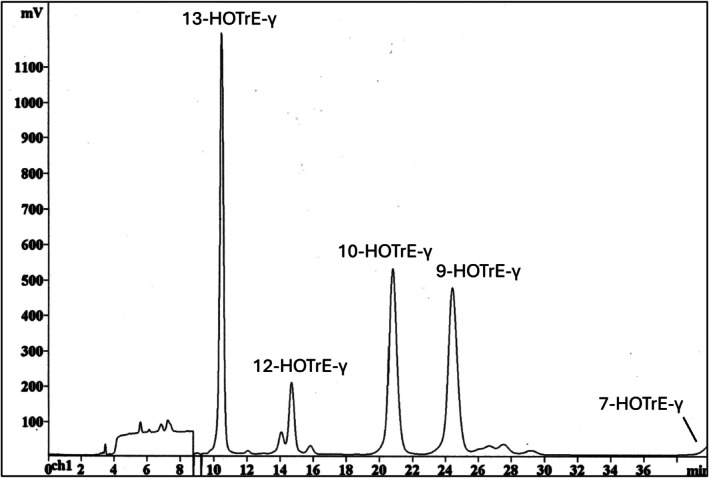
HPLC separation of the mixture of mono‐hydroxylated metabolites of GLA (RI detector).

GLA metabolites eluted between 10.00 and 42.00 min (Scheme 13). The peaks were identified by GC–MS analyses of the purified fractions (Scheme S3). All the GLA metabolites could be obtained with good (> 95%) to very high (> 98%) purities (Table 1).

**SCHEME 14:**
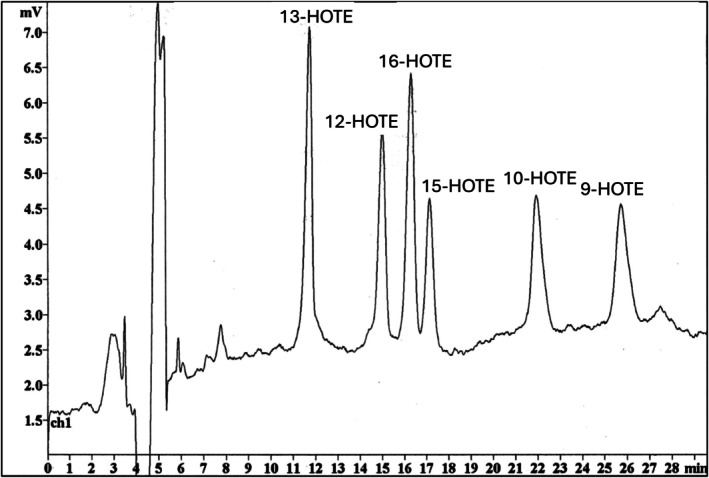
HPLC separation of the mixture of mono‐hydroxylated metabolites of SDA (RI detector).

SDA metabolites eluted between 11.00 and 40.00 min (Scheme 14). As for the previous fatty acids, the peaks were identified by GC–MS analyses of the collected fractions (Scheme S4). Unfortunately, it was discovered after collection that 7‐HOTE eluted much later, between 40 and 42 min. Given that its structure is similar to the other metabolites, this 15‐min delay was unexpected. Moreover, the software window has a maximum of 40 min, so the peak was not detected during the collection process and was lost. With the exception of 9‐HOTE, which coeluted with an unidentified impurity, all the SDA metabolites were obtained with good (> 95%) to very high (> 98%) purities (Table 1).

**SCHEME 15:**
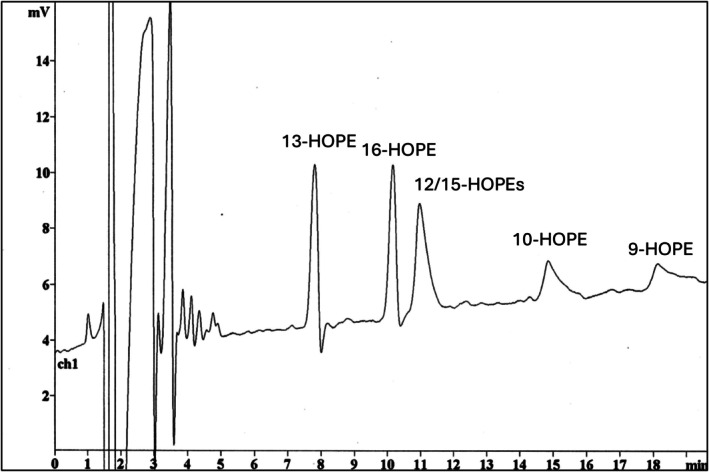
HPLC separation of the mixture of mono‐hydroxylated metabolites of octadecapentaenoic acid (ODPA) (RI detector).

ODPA metabolites eluted between 7.00 and 22.00 min (Scheme 15). The peaks were identified by GC–MS analyses of the collected fractions (Scheme S5). The ODPA metabolites were found to be unstable and needed to be kept on dry ice under argon. Under these storage conditions, 12‐HOPE and 15‐HOPE were obtained as a 60/40 mixture with a purity of 94% and the 10‐ and 13‐isomers could be obtained in very high purities (> 98%). The 9‐isomer partially degraded and could be isolated with a low purity of 80%, and the 16‐isomer completely degraded and could not be isolated. Further studies are therefore required to evaluate the stability of the 9‐ and 16‐HOPE isomers.

## 4 Conclusion

Photosensitized oxidation of PUFAs offers a fast and simple approach for the generation of mono‐hydroxylated oxylipins. In a single synthetic step, multiple metabolites of individual PUFAs could be produced, the majority of which could be successfully separated by chromatography. Metabolites from ALA, GLA, SDA, and OPDA could be obtained with purities from 80 to > 98% and an overall yield from 6.0% to 28.4%. Given that the conversion of every reaction was complete, the low yields can be mainly attributed to the formation of multiple oxygenated products, notably dihydroperoxides giving rise to diols in the reaction products. The number of unsaturations of the parent PUFA affected the stability of the mono‐hydroxylated metabolites, with increased unsaturations resulting in higher formation of degradation products. It is likely that higher purities could be obtained with additional rounds of purification (except for 9‐HOPE, which partially degraded following purification).

For 3 out of the 4 PUFAs studied, not all expected photooxidation products were detected (e.g., 6‐HOTrE‐γ, 6‐HOTE, 3‐, 4‐, and 6‐HOPE). While the underlying reason is not clear, given that the compounds were not detected by GC–MS (while the more polar diols were detected), it is likely that the products were either not formed or quickly degraded. Additional modifications to the method could be beneficial, with future efforts attempting to optimize the procedure to increase yields, decrease byproducts, and determine the reproducibility of the procedure. It would be particularly interesting to test if performing the photooxidation with the methyl esters of the PUFAs would lead to stable formation of the undetected products. In addition, it would be valuable to separate the enantiomers, which we have previously achieved using a chiral column on a SFC system (Quaranta et al. 2022). Given the need for enantioselective biological assays, this could be particularly useful.

This method is useful for increasing the number of available analytical standards to create libraries of new oxylipin metabolites. A drawback of the method is the limited overall yields, especially for SDA and ODPA photooxidations, that render the reactions complicated to scale up and prevent the production of larger amounts of metabolites. It is likely that this approach could be extended to PUFAs of varying chain length, suggesting that photosensitized oxidation could be employed to rapidly prepare hydroperoxides from multiple unsaturated fatty acids. As interest in oxylipins continues to increase, this approach will be useful for preparing multiple standards for the study of these new compounds.

## Author Contributions

**Johanna Revol‐Cavalier:** writing – original draft, methodology, investigation, conceptualization. **Mats Hamberg:** writing – original draft, methodology, investigation, supervision, conceptualization. **Craig E. Wheelock:** writing – review and editing, funding acquisition, supervision, conceptualization.

## Funding

C.E.W. acknowledges support from the Hjärt‐Lungfonden (20230463, 20210519) and Vetenskapsrådet (2022‐00796).

## Conflicts of Interest

The authors declare no conflicts of interest.

## Supporting information


**Scheme S1:** RP‐HPLC separation of 15‐HOTrE and 16‐HOTrE (UV detector).
**Scheme S2:** Chromatogram and mass spectrum of purified ALA metabolites.
**Scheme S3:** Chromatogram and mass spectrum of purified GLA metabolites.
**Scheme S4:** Chromatogram and mass spectrum of purified SDA metabolites.
**Scheme S5:** Chromatogram and mass spectrum of purified ODPA metabolites (chromatogram and mass spectrum of 16‐HOPE were acquired before degradation).
**Scheme S6:** ^1^H NMR spectrum of ODPA methyl ester.

## Data Availability

The data that support the findings of this study are available from the corresponding author upon reasonable request.

## References

[lipd70040-bib-0001] Alberti, M. N. , G. Vassilikogiannakis , and M. Orfanopoulos . 2008. “Stereochemistry of the Singlet Oxygenation of Simple Alkenes: A Stereospecific Transformation.” Organic Letters 10, no. 18: 3997–4000. 10.1021/ol801488w. 18707107

[lipd70040-bib-0002] Derogis, P. B. M. C. , F. P. Freitas , A. S. F. Marques , et al. 2013. “The Development of a Specific and Sensitive LC‐MS‐Based Method for the Detection and Quantification of Hydroperoxy‐ and Hydroxydocosahexaenoic Acids as a Tool for Lipidomic Analysis.” PLoS One 8, no. 10: e77561. 10.1371/journal.pone.0077561. 24204871 PMC3812029

[lipd70040-bib-0003] Fiasella, A. , A. Nuzzi , M. Summa , et al. 2014. “3‐Aminoazetidin‐2‐One Derivatives as N‐Acylethanolamine Acid Amidase (NAAA) Inhibitors Suitable for Systemic Administration.” ChemMedChem 9, no. 7: 1602–1614. 10.1002/cmdc.201300546. 24828120 PMC4224963

[lipd70040-bib-0004] Foote, C. S. 1968. “Mechanisms of Photosensitized Oxidation.” Science 162, no. 3857: 963–970. 10.1126/science.162.3857.963. 4972417

[lipd70040-bib-0005] Frankel, E. N. , W. E. Neff , and T. R. Bessler . 1979. “Analysis of Autoxidized Fats by Gas Chromatography‐Mass Spectrometry: V. Photosensitized Oxidation.” Lipids 14, no. 12: 961–967. 10.1007/BF02533431.

[lipd70040-bib-0006] Frimer, A. A. 1979. “The Reaction of Singlet Oxygen With Olefins: The Question of Mechanism.” Chemical Reviews 79, no. 5: 359–387. 10.1021/cr60321a001.

[lipd70040-bib-0007] Gardner, H. W. , and M. J. Grove . 2001. “Method to Produce 9(S)‐Hydroperoxides of Linoleic and Linolenic Acids by Maize Lipoxygenase.” Lipids 36, no. 5: 529–533. 10.1007/s11745-001-0753-1. 11432467

[lipd70040-bib-0008] Gerwick, W. H. , M. Moghaddam , and M. Hamberg . 1991. “Oxylipin Metabolism in the Red Alga Gracilariopsis Lemaneiformis: Mechanism of Formation of Vicinal Dihydroxy Fatty Acids.” Archives of Biochemistry and Biophysics 290, no. 2: 436–444. 10.1016/0003-9861(91)90563-x. 1929410

[lipd70040-bib-0009] Gorman, A. A. , and M. A. Rodgers . 1992. “Current Perspectives of Singlet Oxygen Detection in Biological Environments.” Journal of Photochemistry and Photobiology. B 14, no. 3: 159–176. 10.1016/1011-1344(92)85095-c. 1432388

[lipd70040-bib-0010] Hamberg, M. 2011. “Stereochemistry of Hydrogen Removal During Oxygenation of Linoleic Acid by Singlet Oxygen and Synthesis of 11(S)‐Deuterium‐Labeled Linoleic Acid.” Lipids 46, no. 2: 201–206. 10.1007/s11745-010-3510-4. 21161604

[lipd70040-bib-0011] Held, A. M. , D. J. Halko , and J. K. Hurst . 1978. “Mechanisms of Chlorine Oxidation of Hydrogen Peroxide.” Journal of the American Chemical Society 100, no. 18: 5732–5740. 10.1021/ja00486a025.

[lipd70040-bib-0012] Iacazio, G. , G. Langrand , J. Baratti , G. Buono , and C. Triantaphylides . 1990. “Preparative, Enzymic Synthesis of Linoleic Acid (13S)‐Hydroperoxide Using Soybean Lipoxygenase‐1.” Journal of Organic Chemistry 55, no. 5: 1690–1691. 10.1021/jo00292a056.

[lipd70040-bib-0013] Lindgren, B. O. , T. Nilsson , S. Husebye , Ø. Mikalsen , K. Leander , and C.‐G. Swahn . 1973. “Preparation of Carboxylic Acids From Aldehydes (Including Hydroxylated Benzaldehydes) by Oxidation With Chlorite.” Acta Chemica Scandinavica 27: 888–890. 10.3891/acta.chem.scand.27-0888.

[lipd70040-bib-0014] Murray, R. W. , and M. L. Kaplan . 1969. “Singlet Oxygen Sources in Ozone Chemistry. Chemical Oxygenations Using the Adducts Between Phosphite Esters and Ozone.” Journal of the American Chemical Society 91, no. 19: 5358–5364. 10.1021/ja01047a027.

[lipd70040-bib-0027] Ngan, F. , and M. Toofan . 1991. “Modification of Preparation of Diazomethane for Methyl Esterification of Environmental Samples Analysis by Gas Chromatography.” Journal of Chromatographic Science 29: 8–10.

[lipd70040-bib-0015] Parchem, K. , S. Letsiou , T. Petan , et al. 2024. “Oxylipin Profiling for Clinical Research: Current Status and Future Perspectives.” Progress in Lipid Research 95: 101276. 10.1016/j.plipres.2024.101276. 38697517

[lipd70040-bib-0016] Peters, J. W. , P. J. Bekowies , A. M. Winer , and J. N. Pitts Jr. 1975. “Potassium Perchromate as a Source of Singlet Oxygen.” Journal of the American Chemical Society 97, no. 12: 3299–3306. 10.1021/ja00845a003.

[lipd70040-bib-0017] Przybyla, D. , C. Göbel , A. Imboden , M. Hamberg , I. Feussner , and K. Apel . 2008. “Enzymatic, but Not Non‐Enzymatic, 1O2‐Mediated Peroxidation of Polyunsaturated Fatty Acids Forms Part of the EXECUTER1‐Dependent Stress Response Program in the Flu Mutant of *Arabidopsis thaliana* .” Plant Journal 54, no. 2: 236–248. 10.1111/j.1365-313X.2008.03409.x. 18182022

[lipd70040-bib-0018] Quaranta, A. , B. Zöhrer , J. Revol‐Cavalier , et al. 2022. “Development of a Chiral Supercritical Fluid Chromatography‐Tandem Mass Spectrometry and Reversed‐Phase Liquid Chromatography‐Tandem Mass Spectrometry Platform for the Quantitative Metabolic Profiling of Octadecanoid Oxylipins.” Analytical Chemistry 94, no. 42: 14618–14626. 36219822 10.1021/acs.analchem.2c02601 PMC9607849

[lipd70040-bib-0019] Revol‐Cavalier, J. , A. Quaranta , J. W. Newman , A. R. Brash , M. Hamberg , and C. E. Wheelock . 2025. “The Octadecanoids: Synthesis and Bioactivity of 18‐Carbon Oxygenated Fatty Acids in Mammals, Bacteria, and Fungi.” Chemical Reviews 125, no. 1: 1–90. 10.1021/acs.chemrev.3c00520. 39680864 PMC11719350

[lipd70040-bib-0021] Steinbeck, M. J. , A. U. Khan , and M. J. Karnovsky . 1992. “Intracellular Singlet Oxygen Generation by Phagocytosing Neutrophils in Response to Particles Coated With a Chemical Trap.” Journal of Biological Chemistry 267, no. 19: 13425–13433. 10.1016/S0021-9258(18)42228-4. 1320020

[lipd70040-bib-0022] Terao, J. , and S. Matsushita . 1981. “The Isomeric Compositions of Hydroperoxides Produced by Oxidation of Arachidonic Acid With Singlet Oxygen.” Agricultural and Biological Chemistry 45, no. 3: 587–593. 10.1271/bbb1961.45.587.

[lipd70040-bib-0023] Thomas, M. J. , and W. A. Pryor . 1980. “Singlet Oxygen Oxidation of Methyl Linoleate: Isolation and Characterization of the NaBH4‐Reduced Products.” Lipids 15, no. 7: 544–548. 10.1007/BF02534228.

[lipd70040-bib-0024] Triantaphylidès, C. , and M. Havaux . 2009. “Singlet Oxygen in Plants: Production, Detoxification and Signaling.” Trends in Plant Science 14, no. 4: 219–228. 10.1016/j.tplants.2009.01.008. 19303348

[lipd70040-bib-0025] Wasserman, H. H. , J. R. Scheffer , and J. L. Cooper . 1972. “Singlet Oxygen Reactions With 9,10‐Diphenylanthracene Peroxide.” Journal of the American Chemical Society 94, no. 14: 4991–4996. 10.1021/ja00769a034.

[lipd70040-bib-0026] Zhang, W. , M. Sun , and R. G. Salomon . 2006. “Preparative Singlet Oxygenation of Linoleate Provides Doubly Allylic Dihydroperoxides: Putative Intermediates in the Generation of Biologically Active Aldehydes In Vivo.” Journal of Organic Chemistry 71, no. 15: 5607–5615. 10.1021/jo0605795. 16839140

