# Supplementary material for: Photooxidation of 18‐Carbon Polyunsaturated Fatty Acids to Prepare Mono‐Hydroxylated Octadecanoids
Source: Lipids. 2026 Feb 17;61(4):447–58. doi: 10.1002/lipd.70040 (PMC13341409; doi:10.1002/lipd.70040)
Supplement: Supplementary file 1 — Scheme S1: RP‐HPLC separation of 15‐HOTrE and 16‐HOTrE (UV detector). Scheme S2: Chromatogram and mass spectrum of purified ALA metabolites. Scheme S3: Chromatogram and mass spectrum of purified GLA metabolites. Scheme S4: Chromatogram and mass spectrum of purified SDA metabolites. Scheme S5: Chromatogram and mass spectrum of purified ODPA metabolites (chromatogram and mass spectrum of 16‐HOPE were acquired before degradation). Scheme S6: 1H NMR spectrum of ODPA methyl ester. [file LIPD-61-447-s001.pdf]

Supplementary information

Photooxidation of 18-carbon polyunsaturated fatty acids to prepare mono-hydroxylated octadecanoids

Johanna Revol-Cavalier[1,2], Mats Hamberg[1,2], Craig E. Wheelock[1,3,*]

[1]Unit of Integrative Metabolomics, Institute of Environmental Medicine, Karolinska Institutet, 171 77, Stockholm, Sweden

[2]Larodan Research Laboratory, Karolinska Institutet, Stockholm, S-171 77, Sweden

[3]Department of Respiratory Medicine and Allergy, Karolinska University Hospital, Stockholm 141-86, Sweden

*corresponding author
Craig E. Wheelock, PhD
Unit of Integrative Metabolomics,
Institute of Environmental Medicine,
Karolinska Institutet,
171 77, Stockholm, Sweden
craig.wheelock@ki.se

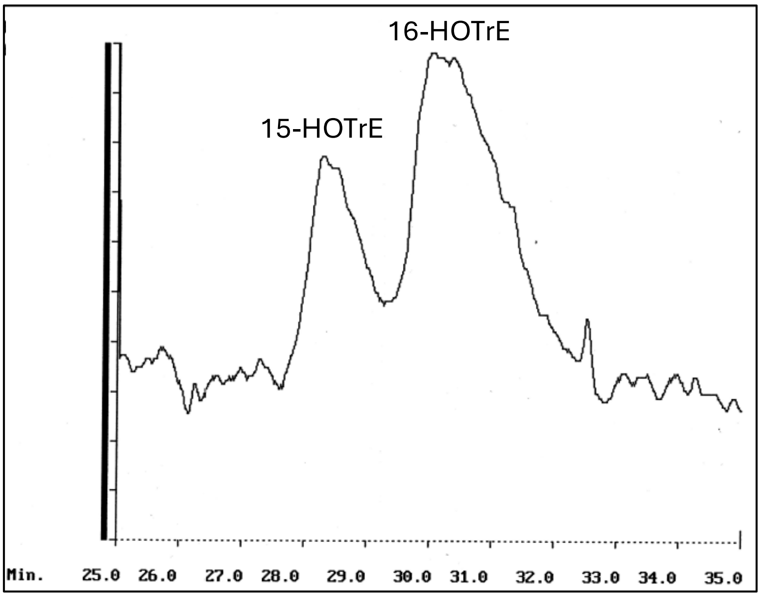

Scheme S1: RP-HPLC separation of 15-HOTrE and 16-HOTrE (UV detector)

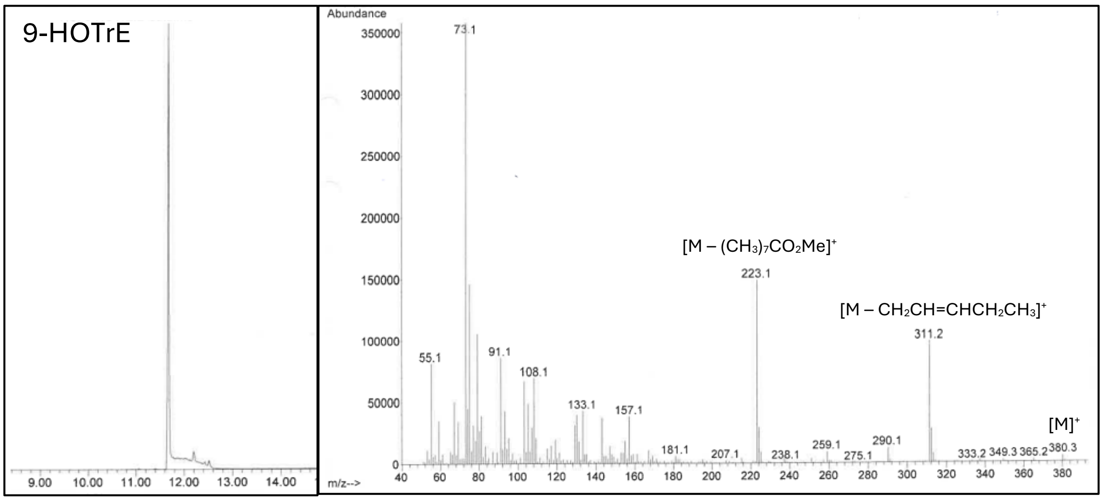
9-HOTrE
Abundance
$[M - (CH_3)_7CO_2Me]^+$
223.1
$[M - CH_2CH{=}CHCH_2CH_3]^+$
311.2
$[M]^+$
380.3
m/z-->

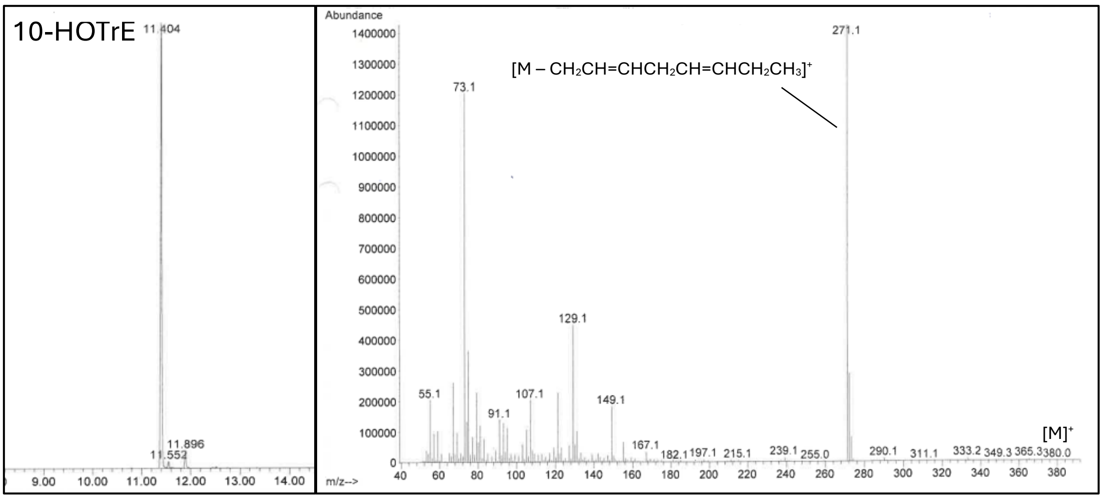
10-HOTrE
11.404
11.896
11.552
Abundance
$[M - CH_2CH{=}CHCH_2CH{=}CHCH_2CH_3]^+$
271.1
$[M]^+$
380.0
m/z-->

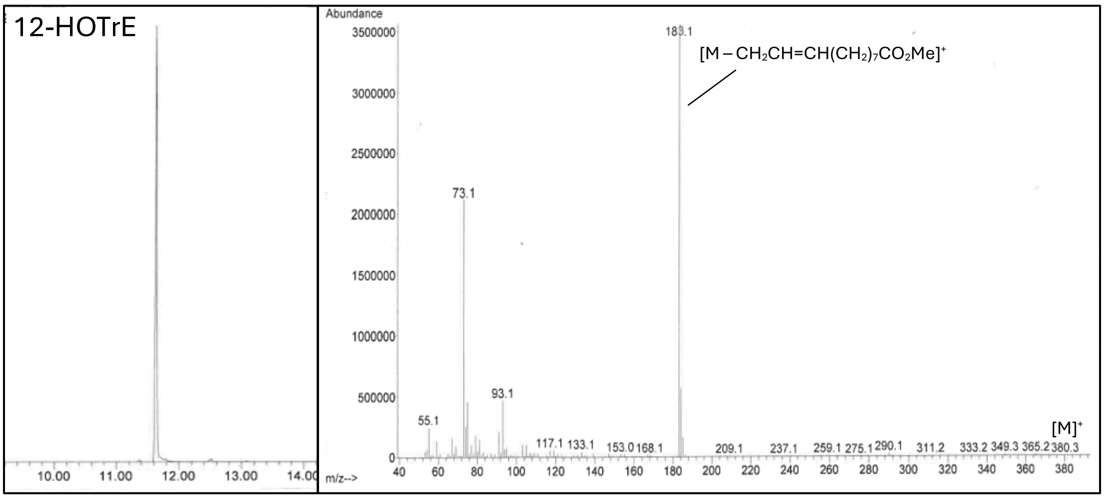
12-HOTrE
Abundance
183.1
$[M - CH_2CH{=}CH(CH_2)_7CO_2Me]^+$
$[M]^+$
380.3
m/z-->

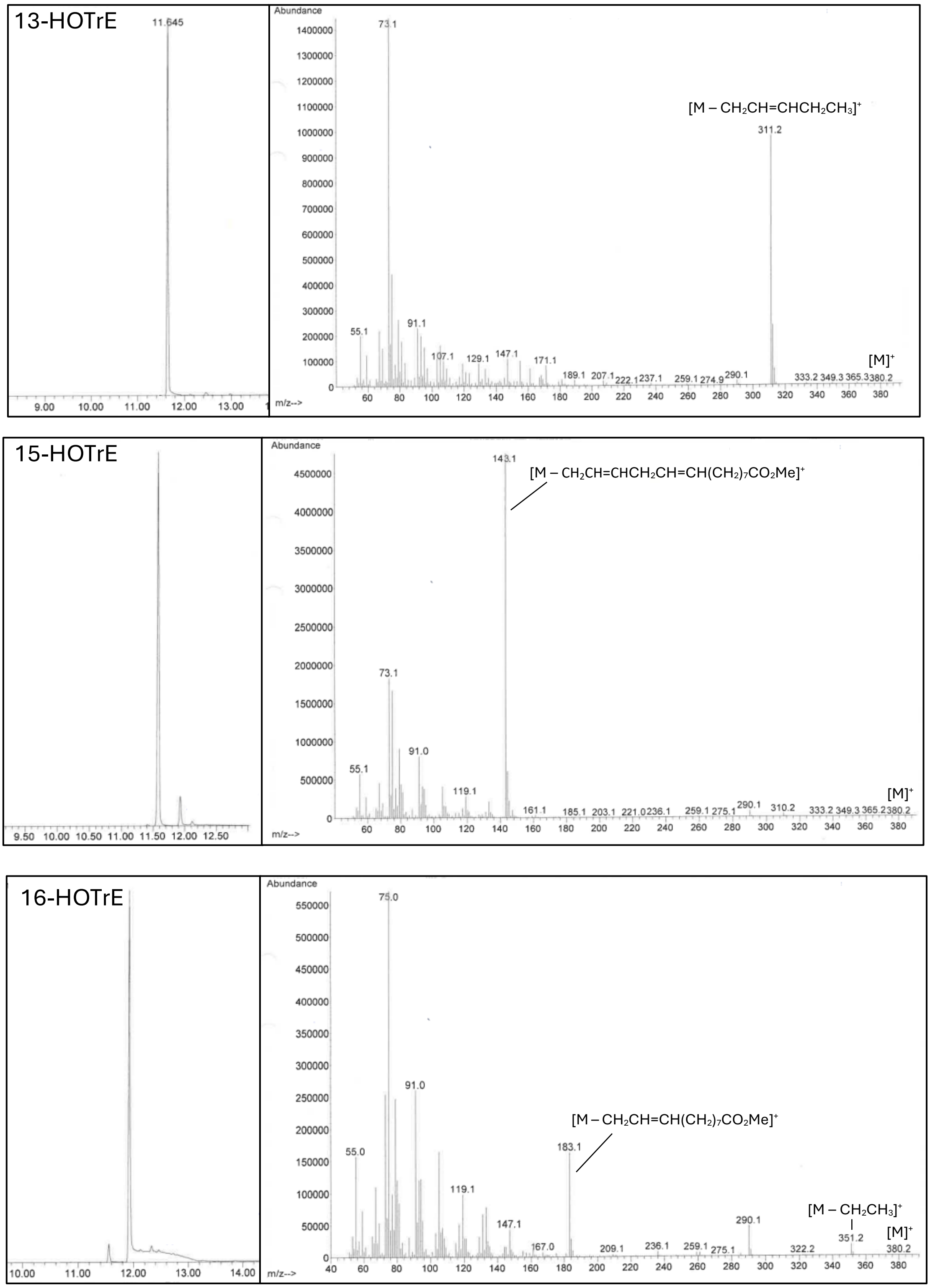

Scheme S2: Chromatogram and mass spectrum of purified ALA metabolites

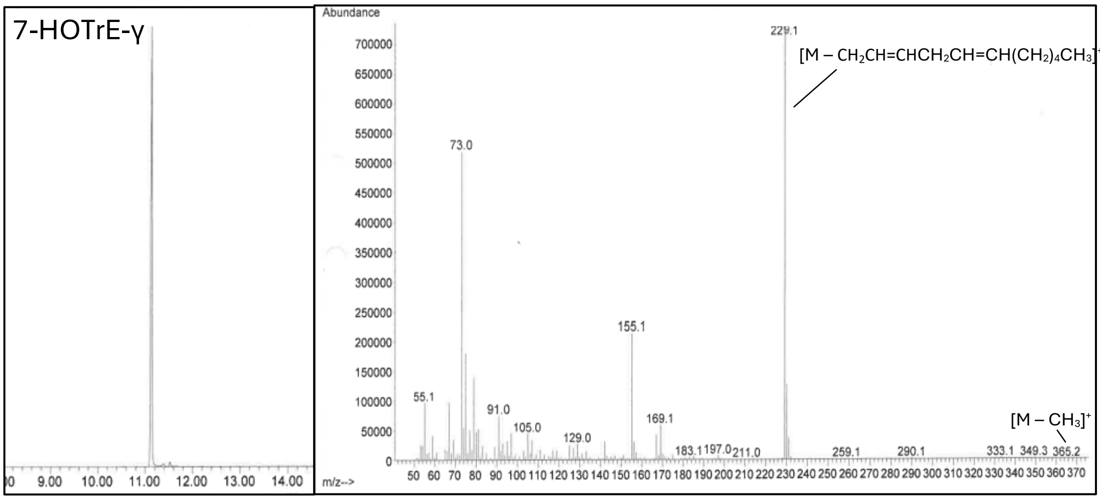
7-HOTrE-γ
Abundance
229.1
$[M - CH_2CH{=}CHCH_2CH{=}CH(CH_2)_4CH_3]^+$
73.0
155.1
55.1
91.0
105.0
129.0
169.1
183.1
197.0
211.0
259.1
290.1
333.1
349.3
365.2
$[M - CH_3]^+$
m/z-->

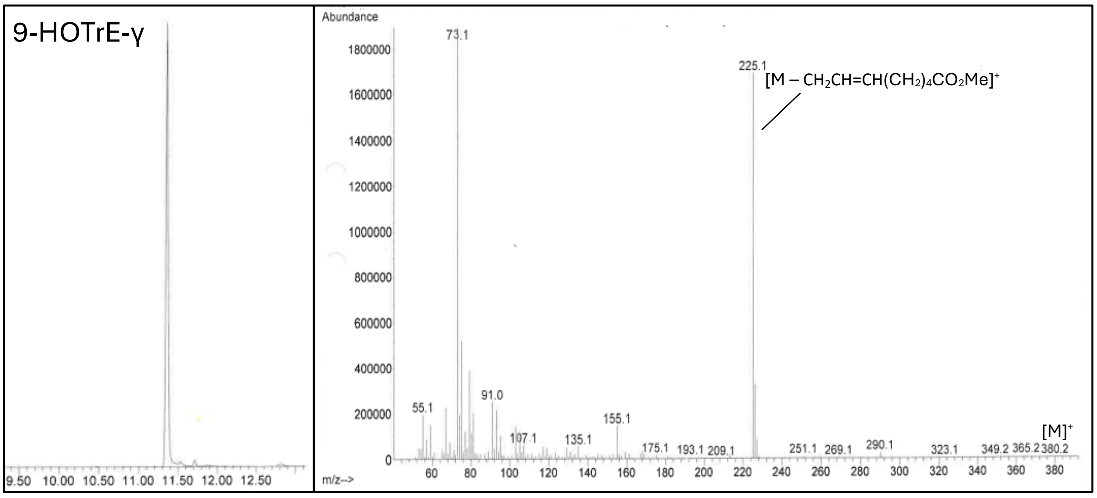
9-HOTrE-γ
Abundance
73.1
225.1
$[M - CH_2CH{=}CH(CH_2)_4CO_2Me]^+$
55.1
91.0
107.1
135.1
155.1
175.1
193.1
209.1
251.1
269.1
290.1
323.1
349.2
365.2
380.2
$[M]^+$
m/z-->

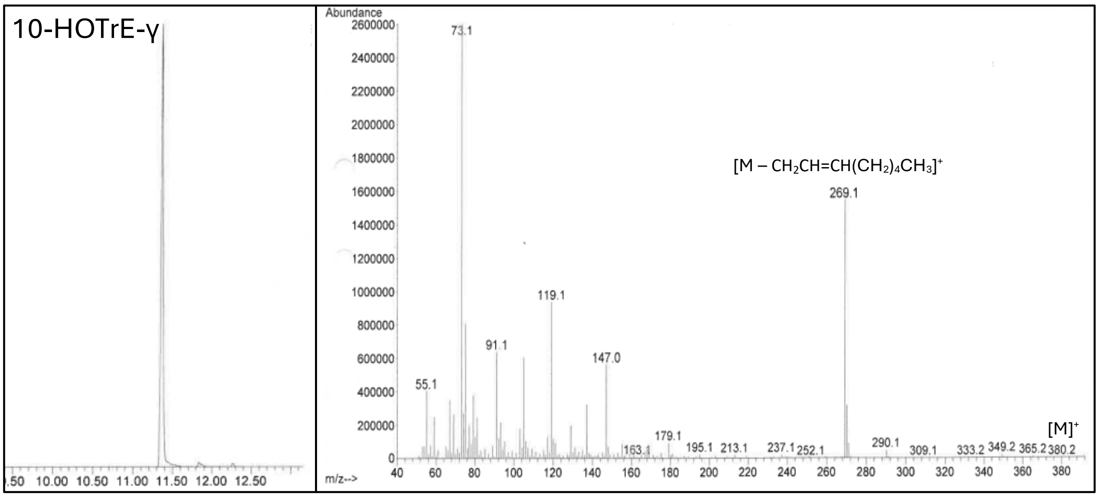
10-HOTrE-γ
Abundance
73.1
$[M - CH_2CH{=}CH(CH_2)_4CH_3]^+$
269.1
119.1
91.1
147.0
55.1
163.1
179.1
195.1
213.1
237.1
252.1
290.1
309.1
333.2
349.2
365.2
380.2
$[M]^+$
m/z-->

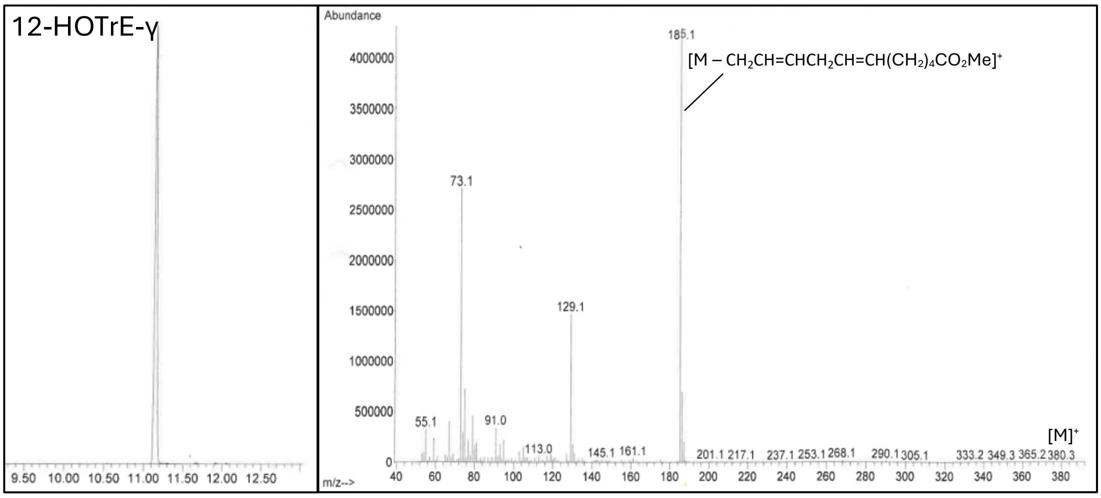

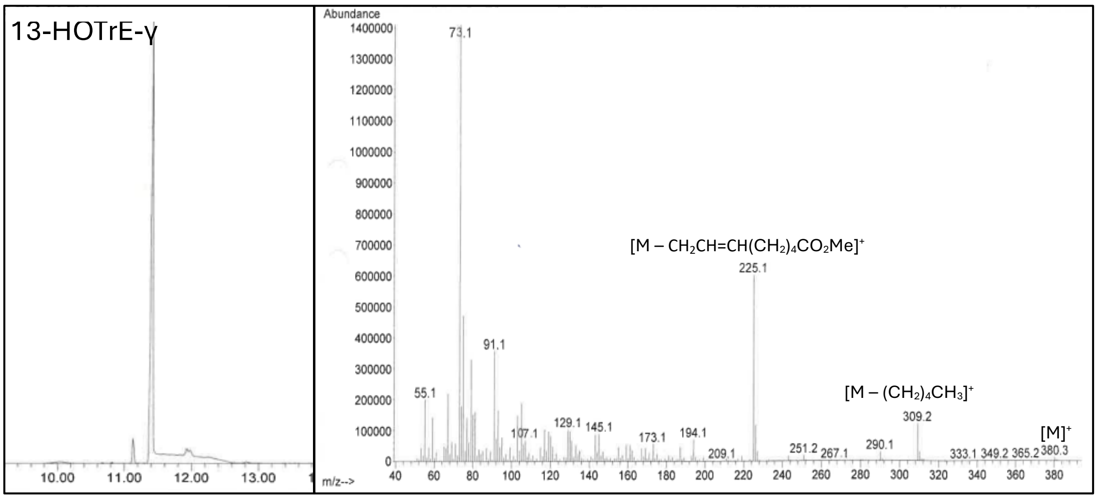

Scheme S3: Chromatogram and mass spectrum of purified GLA metabolites

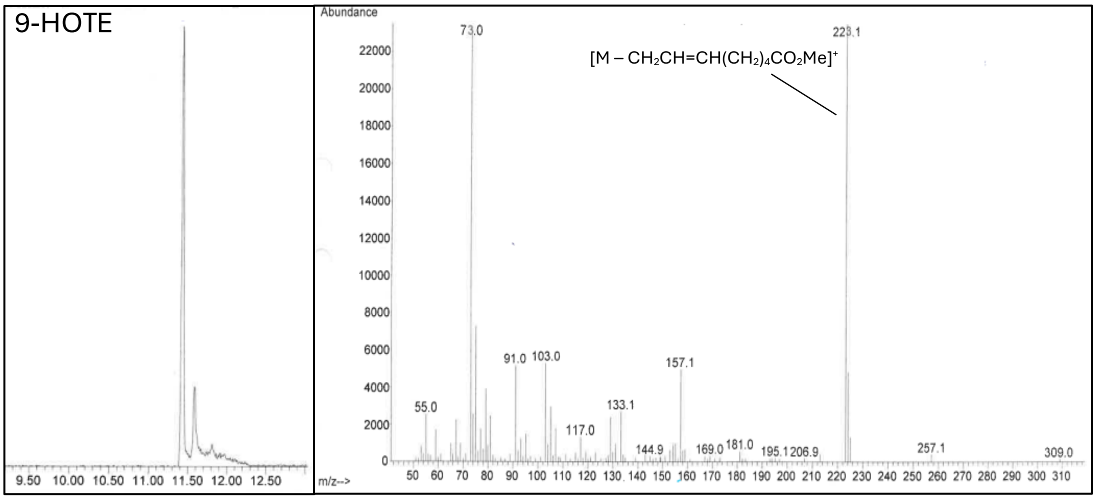
9-HOTE
Abundance
[M – CH2CH=CH(CH2)4CO2Me]+
73.0
228.1
91.0
103.0
157.1
55.0
133.1
117.0
144.9
169.0
181.0
195.1
206.9
257.1
309.0
m/z-->

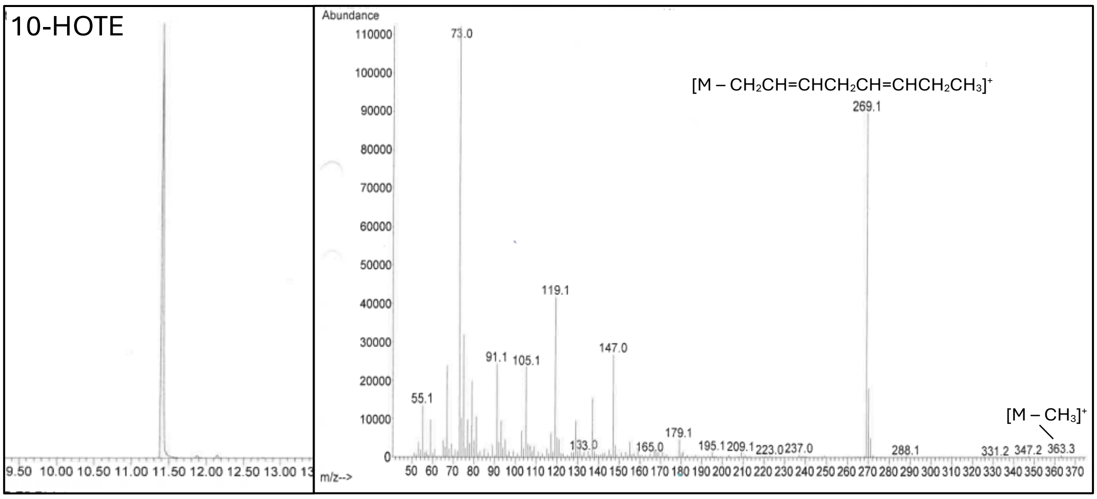
10-HOTE
Abundance
73.0
[M – CH2CH=CHCH2CH=CHCH2CH3]+
269.1
119.1
147.0
91.1
105.1
55.1
[M – CH3]+
179.1
133.0
165.0
195.1
209.1
223.0
237.0
288.1
331.2
347.2
363.3
m/z-->

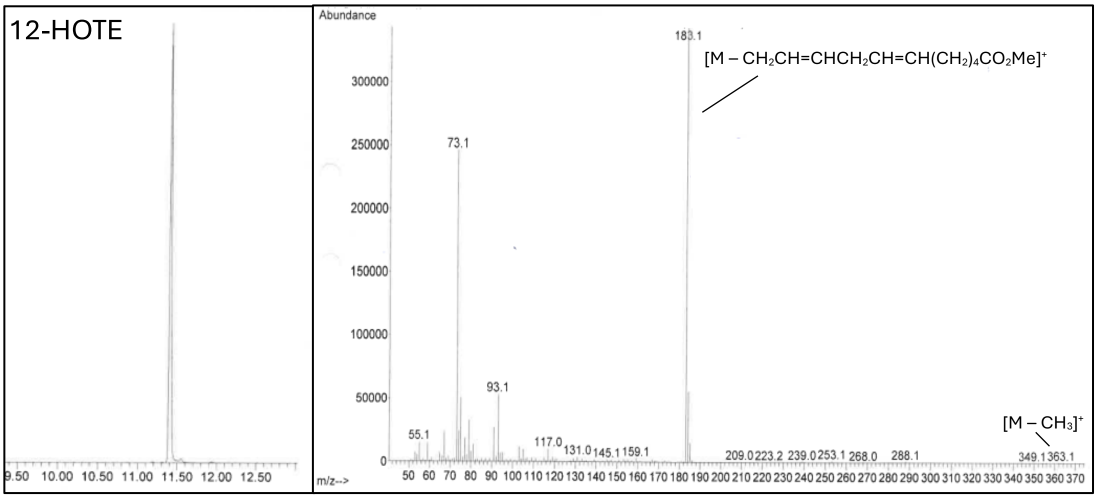
12-HOTE
Abundance
183.1
[M – CH2CH=CHCH2CH=CH(CH2)4CO2Me]+
73.1
93.1
[M – CH3]+
55.1
117.0
131.0
145.1
159.1
209.0
223.2
239.0
253.1
268.0
288.1
349.1
363.1
m/z-->

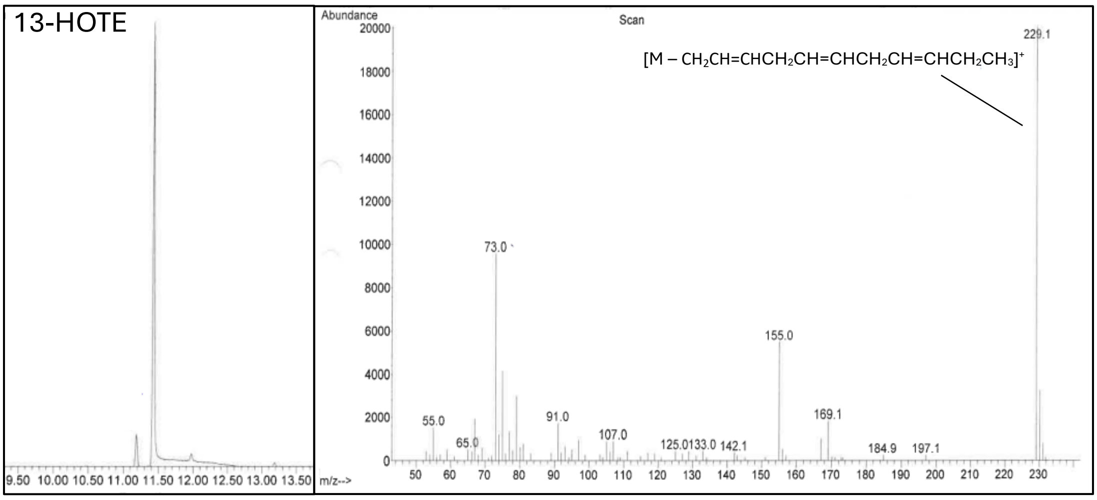

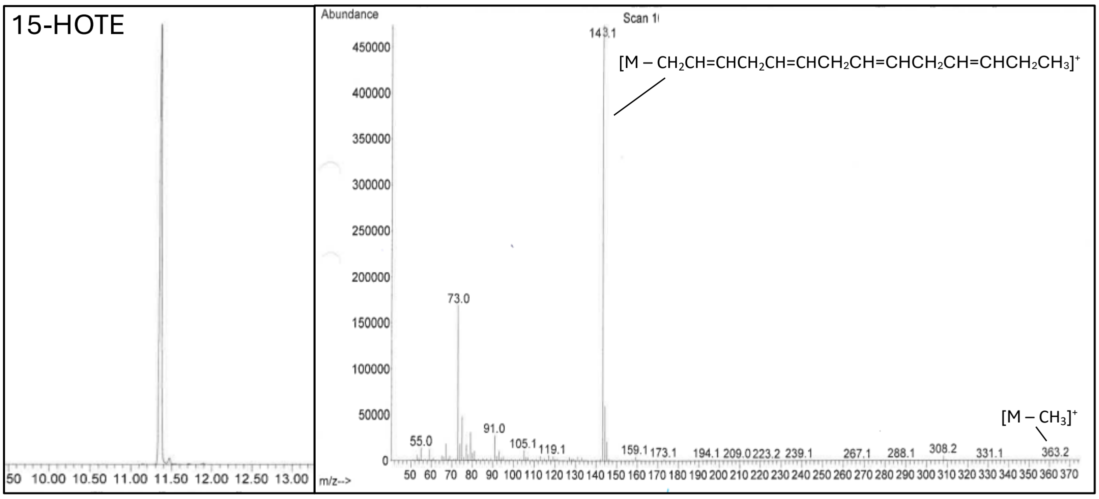

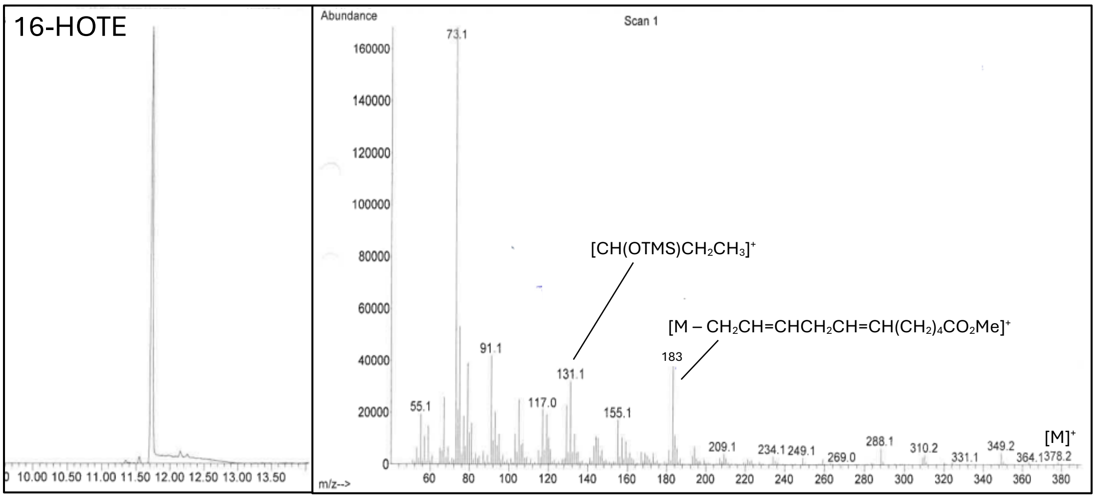

Scheme S4: Chromatogram and mass spectrum of purified SDA metabolites

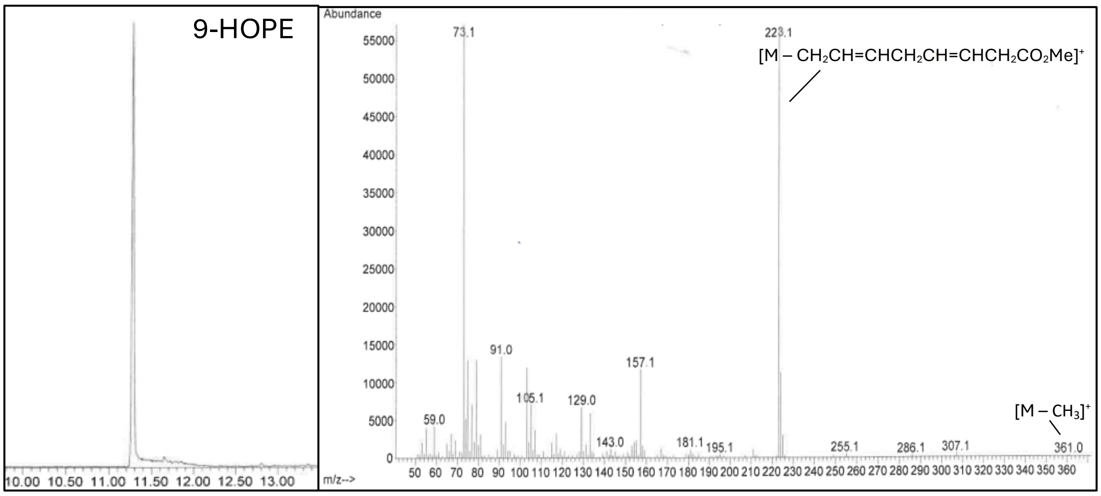
9-HOPE
Abundance
[M – CH₂CH=CHCH₂CH=CHCH₂CO₂Me]⁺
[M – CH₃]⁺
m/z-->
73.1
223.1
91.0
105.1
129.0
157.1
59.0
143.0
181.1
195.1
255.1
286.1
307.1
361.0

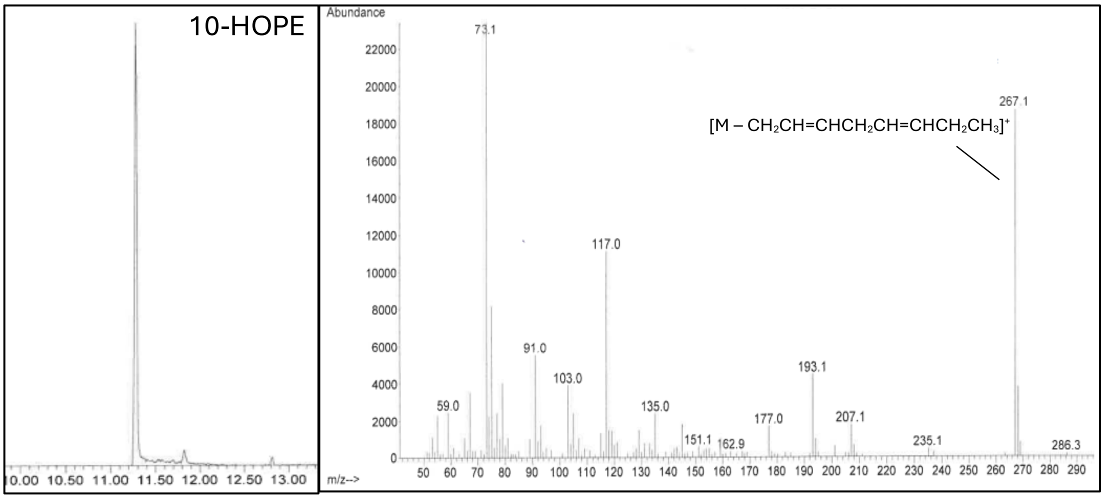
10-HOPE
Abundance
[M – CH₂CH=CHCH₂CH=CHCH₂CH₃]⁺
m/z-->
73.1
267.1
117.0
91.0
103.0
193.1
59.0
135.0
177.0
207.1
151.1
162.9
235.1
286.3

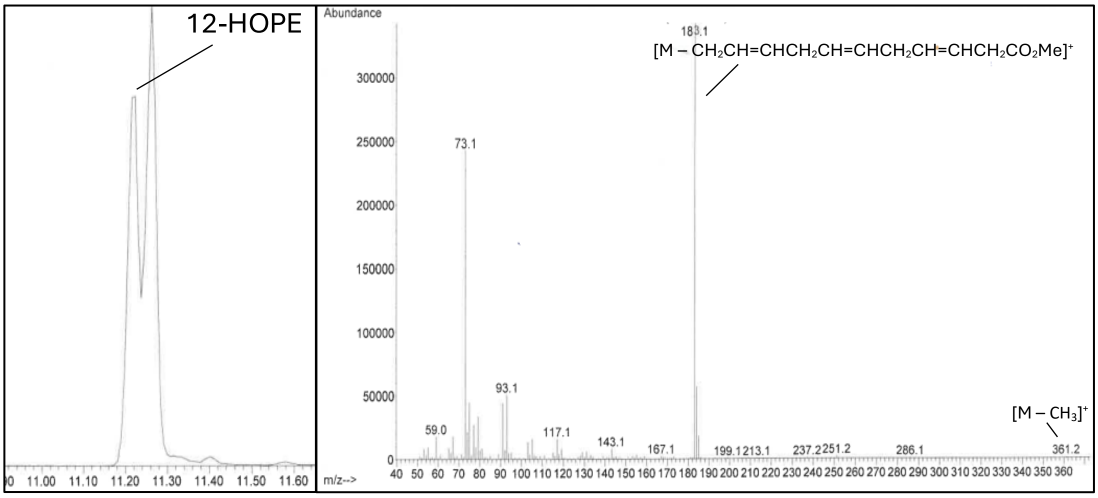
12-HOPE
Abundance
[M – CH₂CH=CHCH₂CH=CHCH₂CH=CHCH₂CO₂Me]⁺
[M – CH₃]⁺
m/z-->
183.1
73.1
93.1
59.0
117.1
143.1
167.1
199.1
213.1
237.2
251.2
286.1
361.2

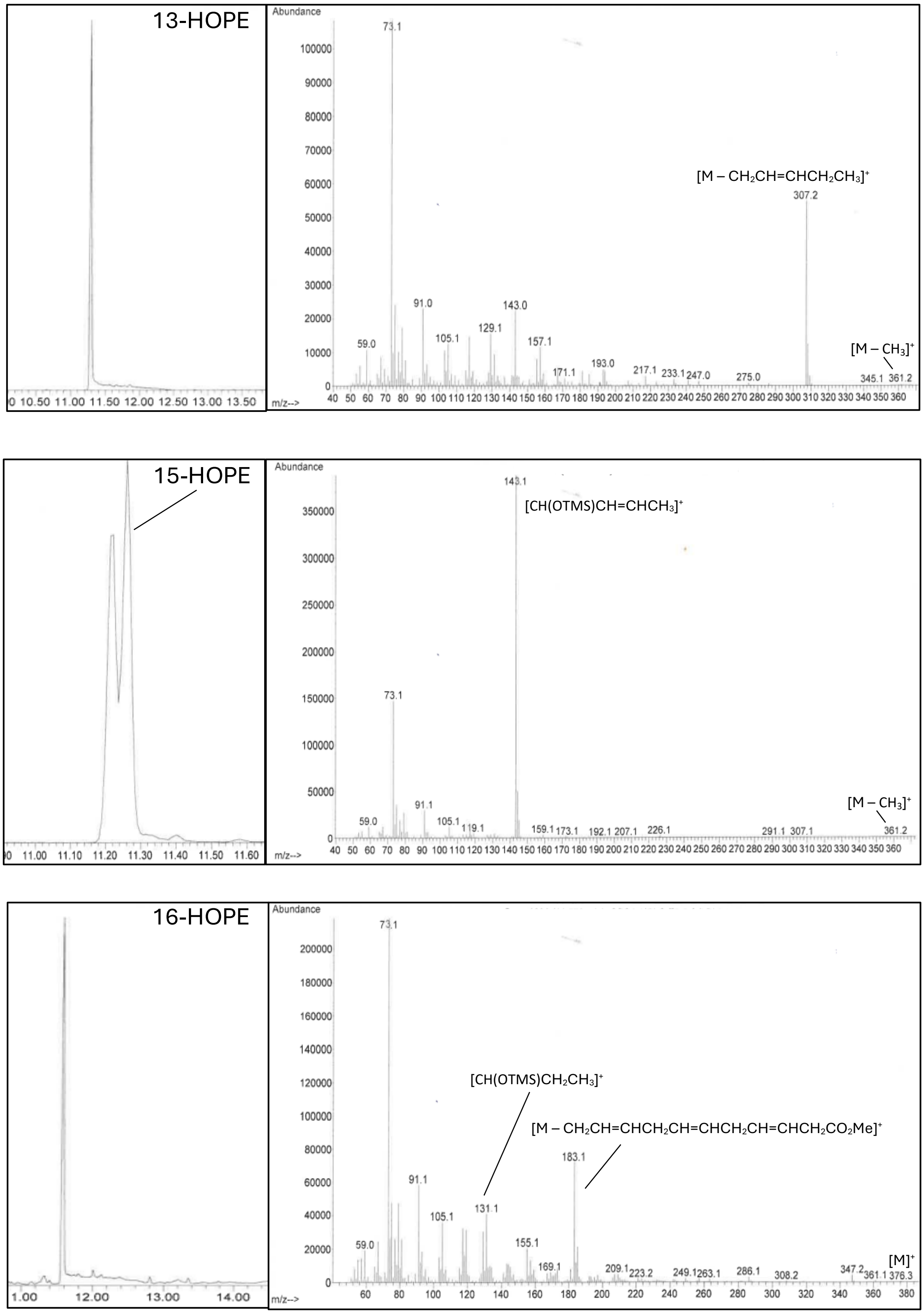

Scheme S5: Chromatogram and mass spectrum of purified ODPA metabolites (chromatogram and mass spectrum of 16-HOPE were acquired before degradation)

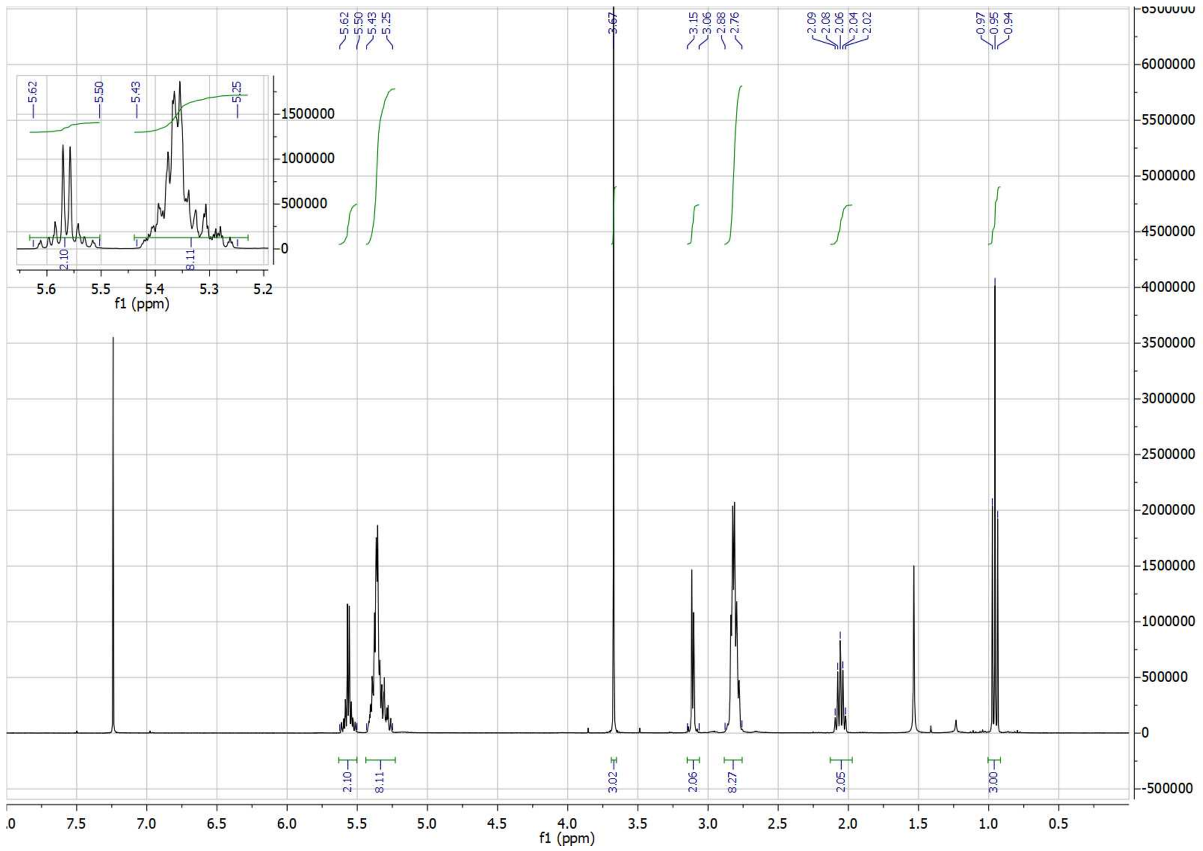

Scheme S6: ^{1}H NMR spectrum of ODPA methyl ester
